# Structural characterization of protective non-neutralizing antibodies targeting Crimean-Congo hemorrhagic fever virus

**DOI:** 10.1038/s41467-022-34923-0

**Published:** 2022-11-26

**Authors:** Ian A. Durie, Zahra R. Tehrani, Elif Karaaslan, Teresa E. Sorvillo, Jack McGuire, Joseph W. Golden, Stephen R. Welch, Markus H. Kainulainen, Jessica R. Harmon, Jarrod J. Mousa, David Gonzalez, Suzanne Enos, Iftihar Koksal, Gurdal Yilmaz, Hanife Nur Karakoc, Sanaz Hamidi, Cansu Albay, Jessica R. Spengler, Christina F. Spiropoulou, Aura R. Garrison, Mohammad M. Sajadi, Éric Bergeron, Scott D. Pegan

**Affiliations:** 1 Department of Pharmaceutical and Biomedical Sciences, College of Pharmacy, University of Georgia, Athens, GA USA; 2 Division of Clinical Care and Research, Institute of Human Virology, University of Maryland School of Medicine, Baltimore, MD USA; 3 Viral Special Pathogens Branch, Division of High-Consequence Pathogens and Pathology, Centers for Disease Control and Prevention, Atlanta, GA USA; 4 Division of Biomedical Sciences, University of California Riverside, Riverside, CA USA; 5 Virology Division, United States Army Medical Research Institute of Infectious Diseases, Fort Detrick, MD USA; 6 Department of Infectious Diseases, University of Georgia College of Veterinary Medicine, Athens, GA 30602 USA; 7 Center for Vaccines and Immunology, University of Georgia College of Veterinary Medicine, Athens, GA 30602 USA; 8 Department of Infectious Disease and Clinical Microbiology, Acibadem University Atakent Hospital, Istanbul, Turkey; 9 Department of Infectious Diseases, Karadeniz Technical University School of Medicine, Trabzon, Turkey; 10 Department of Infectious Disease and Clinical Microbiology, Bitlis State Hospital, Bitlis, Turkey; 11 Department of Chemistry & Life Science, United States Military Academy, West Point, NY USA

**Keywords:** X-ray crystallography, Virus-host interactions, Antibodies

## Abstract

Crimean-Congo Hemorrhagic Fever Virus (CCHFV) causes a life-threatening disease with up to a 40% mortality rate. With no approved medical countermeasures, CCHFV is considered a public health priority agent. The non-neutralizing mouse monoclonal antibody (mAb) 13G8 targets CCHFV glycoprotein GP38 and protects mice from lethal CCHFV challenge when administered prophylactically or therapeutically. Here, we reveal the structures of GP38 bound with a human chimeric 13G8 mAb and a newly isolated CC5-17 mAb from a human survivor. These mAbs bind overlapping epitopes with a shifted angle. The broad-spectrum potential of c13G8 and CC5-17 and the practicality of using them against Aigai virus, a closely related nairovirus were examined. Binding studies demonstrate that the presence of non-conserved amino acids in Aigai virus corresponding region prevent CCHFV mAbs from binding Aigai virus GP38. This information, coupled with in vivo efficacy, paves the way for future mAb therapeutics effective against a wide swath of CCHFV strains.

## Abstract (web summary)

There are currently no approved treatments for Crimean-Congo Hemorrhagic Fever Virus (CCHFV) infection. In this study, the authors structurally characterize the epitope targeted by protective non-neutralizing mouse and human antibodies and provide insights into their broad range potential against various CCHFV strains.

## Introduction

Crimean-Congo Hemorrhagic Fever Virus (CCHFV), a tick-borne virus from the *Nairoviridae* family, causes hemorrhagic fever and death in 5–40% of cases^1,2^. CCHFV is found throughout the Eastern hemisphere, and because of its propensity to spread, WHO has outlined CCHFV as a salient public health risk lacking approved vaccines and therapeutics^1^. Strains of CCHFV are classified into five clades: I–III (endemic in Africa), IV (Asia), V (Europe)^3,4^. A sixth clade no longer remains due to the reclassification of former CCHFV genogroup IV strains into the species Aigai virus, which rarely cause severe illness^3^. CCHFV possesses a negative sense tri-segmented RNA genome, consisting of a Large (L), Medium (M), and Small (S) segment^1,5,6^. Most therapeutic and vaccine development efforts have focused on utilizing proteins derived from the M-segment encoded glycoprotein precursor (GPC). The GPC includes two structural glycoproteins (G_N_ and G_C_) and secreted glycoprotein 38 (GP38) containing proteins that also can be linked to a mucin-like domain (MLD) and one non-structural protein (Nsm)^7–14^.

For CCHFV, therapeutic development of neutralizing mAbs against the structural proteins G_C_ or G_N_ has proven difficult. Anti-G_C_ mAbs have demonstrated prophylactic efficacy but need to be provided within 30 min post-infection to confer therapeutic efficacy against a CCHFV human isolate from Turkey^8^. In comparison, 13G8 mAb binds to the GP38 region of the GPC, which is of particular interest after demonstrating non-neutralizing protection in a CCHFV IFNAR^−/−^ mouse model both prophylactically and post-challenge^9^. 13G8 has also shown prophylactic potential against strains IbAr10200(Clade III), Afg-09(Clade IV), and Turkey-2004(Clade V)^8,10,15^.

The role of GP38 in viral replication and pathogenesis is still being elucidated, but GP38 is essential in the maturation of CCHFV particles^15^. Additional studies have observed that GP38 may be secreted or localized to the outside of infected cells and the viral envelope^11^. Recently, a DNA vaccine encoding GP38 illustrated that this protein could provide partial protection^16^. While the role of GP38 is not fully understood, there is speculation that GP38 is still attached to the G_N_ and does not originate from gene duplication event^14^. One X-ray structure of GP38 from IbAr10200 has been solved, which has given initial insight into its overall fold, but a GP38-antibody complex has yet to be described^17^. The lack of a molecular basis for how protective non-neutralizing mAbs bind to GP38 has hindered further development of viable broad-spectrum anti-GP38 mAbs.

In this study, a structure of clinically relevant strain of GP38 alone and in complex with mAb human chimeric 13G8 (c13G8) was obtained. Next, seven human anti-GP38 mAbs were isolated from a CCHFV survivor and, in doing so, identified two new antigenic sites on GP38. The complex of GP38 with one of the human-derived mAbs, CC5-17, was also solved. CC5-17 and c13G8 compete at the same antigenic site, but CC5-17 binds free GP38 with much higher affinity. Key amino acid sites responsible for the mAbs' robust affinity across the five phylogenetic clades of CCHFV, but not that of Aigai virus, a closely related nairovirus species were identified. To explore whether the higher affinity of CC5-17 relative to c13G8 equated to greater protection, in vivo studies were performed revealing that c13G8 is more efficacious than the higher affinity CC5-17. This structural information, coupled with efficacy data, further characterizes GP38 as an antigen of interest for vaccination studies while also advancing mAb development toward CCHFV.

## Results

### 13G8 has different affinities toward CCHFV GP38s and closely related Aigai virus

**Fig. 1:**
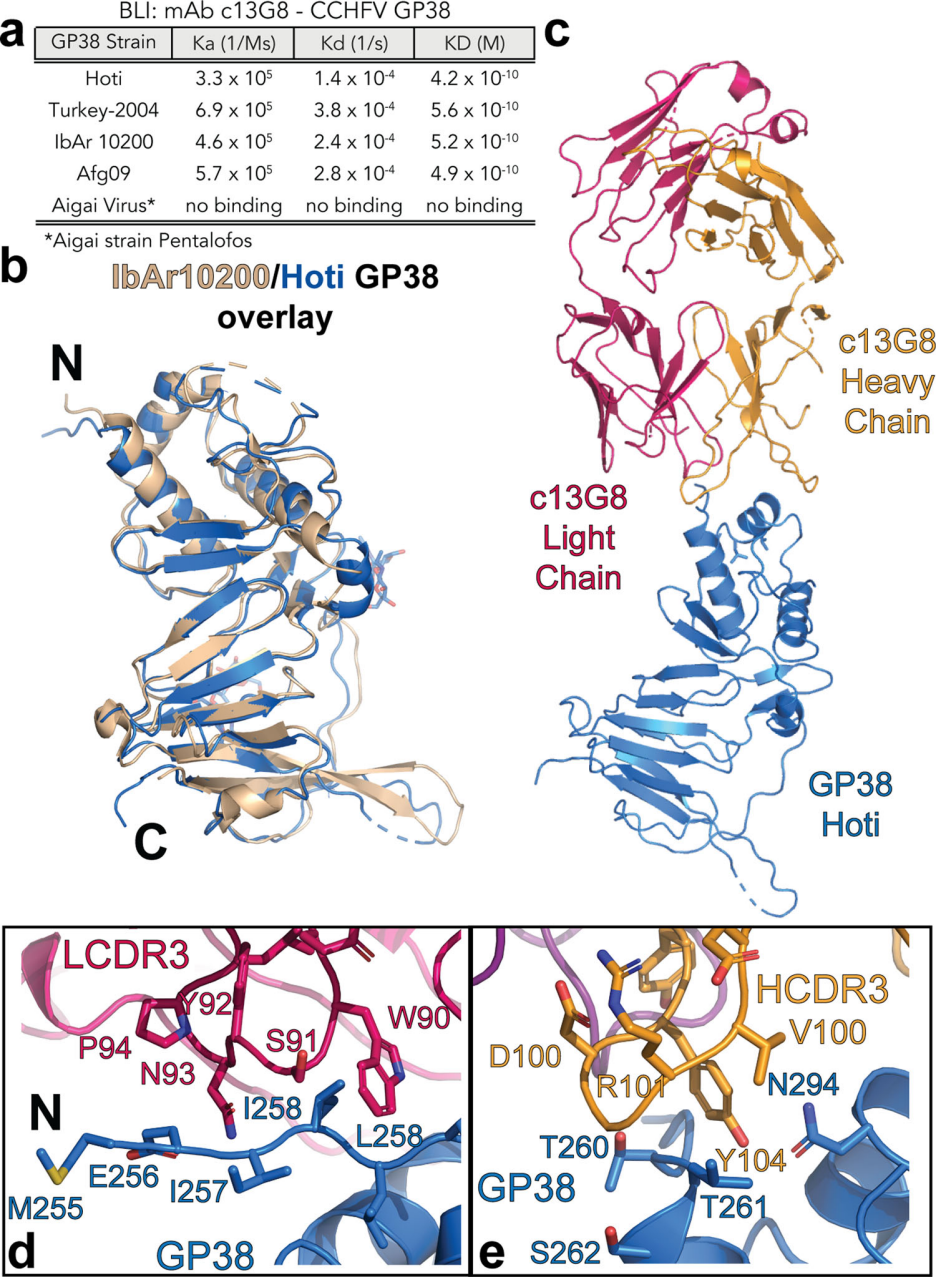
c13G8 interactions with nairovirus GP38. **a** BLI binding kinetics of c13G8 with nairovirus GP38s. **b** Overlay of CCHFV IbAr10200 GP38 (PDBID 6VKF) (wheat) with our solved CCHFV Hoti GP38 Structure (PDBID 8DC5) (blue) **c**, c13G8 Fab complexed with GP38 Hoti **d**, LCDR3 (red) interaction with Hoti GP38 (blue) **e**, HCDR3 (orange) interaction with Hoti GP38 (blue).

To explore the impact of up to 20% difference in amino acid sequence between CCHFV strains, BioLayer Interferometry (BLI) was used to measure the binding affinities of c13G8 against GP38 from CCHFV strains IbAr10200, Afg09, Turkey-2004, and Kosovo/Hoti (Hoti). A similar sub-nanomolar affinity was observed amongst other Clades III–V members (Fig. 1a). To explore if c13G8 had broad-spectrum potential for other nairoviruses, c13G8 was tested against the Aigai virus strain Pentalofos, the closest relative of CCHFV. Interestingly, binding was completely abolished and could not be detected via BLI when c13G8 encountered GP38 from Aigai virus (Fig. 1a).

### Structure of a GP38 from CCHFV strain Hoti

With c13G8 showing low nanomolar or better affinity toward various CCHFV strains but not those of the Aigai virus, the molecular factors behind this phenomenon were sought out. As only one structure of GP38 had been previously solved and was from clade III CCHFV IbAr10200, a highly laboratory-passaged strain with little clinical relevance, we solved an additional structure of GP38 from Clade V CCHFV Hoti. This human isolate is one of the two that can be used to elicit human disease-like symptoms in a non-human primate model^18–23^. In addition, CCHFV Hoti has a 15% sequence identity difference with that of the IbAr10200 isolate that might reveal any major differences in tertiary structures or surface composition between CCHFV GP38s. Crystals of the CCHFV Hoti GP38 were readily obtained, and the structure was solved to a resolution of 3.2 Å (Fig. 1b, Supplementary Table 1). Both the structures of GP38 originating from the IbAr10200 and Hoti strains followed the same global fold. However, an additional alpha helix is detected between Hoti GP38 beta strands 2 and 3 near the N-glycosylation sites (Supplementary Fig 2)^24,25^. Also, a divergent loop conformation is observed in amino acid region 490–500, and like the IbAr10200 structure no electron density for the amino acid region 330–345.

### Structure of c13G8 with GP38 Hoti complex

With no significant tertiary structural differences between GP38 originating from the IbAr10200 and Hoti strains, a crystal structure of Hoti GP38 in complex with the c13G8 Fab was determined to 3.6 Å. (Fig. 1c, Supplementary Table 1). The electron density around the binding interface is well-defined, and the epitope can be identified (Supplementary Fig 3a). A surface area of 783.8 Å^2^ was calculated for the c13G8-GP38 interface^26^. Overall, c13G8 engages with the alpha-helical bundle of GP38 that is characterized by two main components. The first is the LCDR3 that engages and stabilizes the GP38 Hoti N-terminus tail (Fig. 1d). The second component revolves around the interaction of HCDR3 with the GP38 α-helix 2 and fits within a larger hydrophobic pocket in GP38 (Fig. 1e).

### Isolation of anti-GP38 mAbs from CCHF survivors

**Fig. 2:**
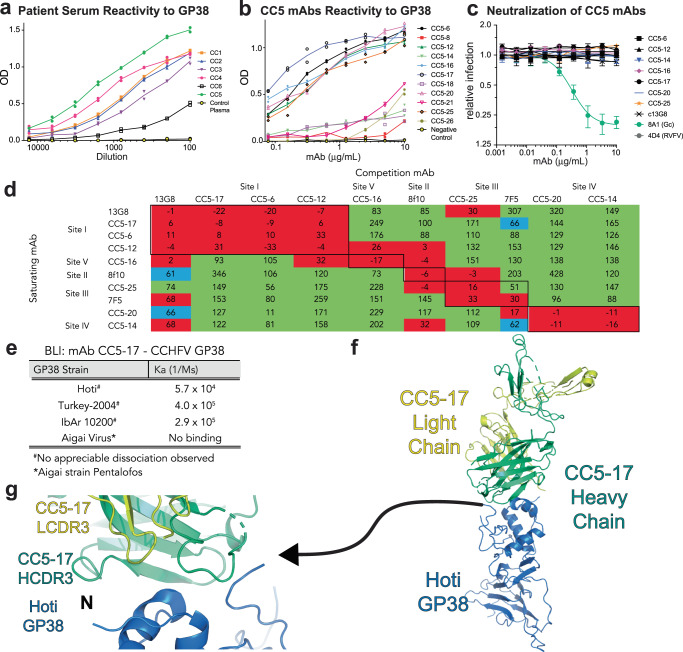
Generation of human-derived anti-GP38 mAb**s**. **a** Six CCHF survivors in Trabzon (Turkey) were recruited, and their plasma were collected to determine their ELISA titers against GP38 Turkey2004. **b** Patient CC5 was selected, and its plasma was used to mine 11 mAbs which were then retested against GP38. ELISA experiments were performed in duplicate (*n* = 2.) **c** Neutralization potential of anti-GP38 mAbs. Foci reduction neutralization assays were performed with CCHFV-ZsG. These data represent one independent experiment out of 2. The average values of replicate wells (*n* = 4) are plotted, and error bars represent the associated standard deviation of the replicates. **d** Seven anti-GP38 CC5 antibodies were then tested against known Group 1, Group 2, and Group 3 antibodies. <33% binding indicates competition (red), 34–66% indicate partial competition (blue), >67% indicate no competition (green). **e** BLI binding kinetics of CC5-17 with nairovirus GP38s **f**, CC5-17 Fab (Yellow and Green) complexed with GP38 Hoti (Blue) (PDBID 8DDK) **g**, HCDR3 (Green) binding and LCDR3 (Yellow) binding interactions with GP38 strain Hoti (blue).

Previously only two mouse anti-GP38 mAbs were tested for in vivo efficacy, 13G8, and 10E11^11,17^. Although 13G8 and 10E11 were found to compete at the same antigenic site, only 13G8 provided protection against CCHFV. The lack of protection in 10E11 was attributed to a lower binding affinity^11^. To explore if human mAbs could provide an alternative to these mouse mAbs, CCHF patients were recruited from Turkey, the country with the most reported annual cases^27–30^. To determine the antibody repertoire against CCHFV GP38 in human survivors, PBMCs and plasma were obtained from six verified survivors of CCHFV infection. Plasma was evaluated by ELISA to determine overall anti-GP38 reactivity against Turkey-2004 GP38 (Fig. 2a). The highest reactivity was seen in donor CC5.

The donor CC5 antibody repertoire was subsequently analyzed through a transcriptomic and genomic approach^31^. Antibody isolation involved affinity purification of the IgG, enzyme digestion of the antibodies, and mass spectrometry of the peptide fragments. These peptide data were reconciled to a patient-specific database from their memory B cells. Of the 11 antibodies produced, seven efficiently bound to Turkey-2004 GP38 (Fig. 2b). Amongst the isolated mAbs, there is relatively low somatic hypermutation (SHM) (<14%), with overall higher mutation rates within the heavy chain (Supplementary Table 2).

### Characterization of human-derived anti-GP38 mAbs

Following the reconciliation and initial ELISA binding characterization of mAbs from donor CC5, binding affinity toward Hoti GP38 was determined using SPR. In comparison to c13G8, which had a KD of 10^−9^ via SPR, the CC5 mAbs either matched or had a higher binding affinity. This included measuring little to no measurable dissociation of the CC5-17 and CC5-6 with GP38. Hence, the overall best binders are CC5-6, CC5-16, and CC5-17 (Table 1). GP38 mAbs from CC5 were also tested for in vitro neutralizing activity against authentic CCHFV producing zsGreen fluorescent protein in infected cells. GP38 mAbs previously isolated from mice were exclusively non-neutralizing as opposed to several anti-Gc mAbs, efficiently neutralizing CCHFV^9,11^. The human derived anti-GP38 mAbs also failed to neutralize CCHFV like their mouse counterparts, suggesting that antibody response toward GP38 is mainly or exclusively non-neutralizing in comparison to the neutralizing anti-Gc mAb 8A1 (Fig. 2c). Next, CC5 mAbs were characterized via the epitope they target. A BLI competition assay was used to test how CC5 mAbs competed against mAbs of known to compete for site I, site II, and site III, represented by mouse-derived antibodies 13G8, 8F10, and 7F5, respectively. Of the seven mAbs isolated, three directly competed with 13G8, which were CC5-17, CC5-6, and CC5-12. CC5-16 was distinctively bound between sites I and II and was thus classified as a site V mAb. CC5-25 directly competed with the site III antibody 7F5. Lastly, the mAbs CC5-14 and CC5-20 only directly competed against each other, identifying a new antigenic site referred to as site IV (Fig. 2d).

**Table 1:** SPR binding characterization of anti-GP38 mAbs against Hoti GP38

mAb	K_a_ (1/Ms)	K_d_ (1/s)	KD (M)
c13G8	1.7 × 10^6^	2.9 × 10^−3^	1.7 × 10^−9^
CC5-17	3.9 × 10^5^	n.m.	n.m.
CC5-16	8.7 × 10^6^	3.1 × 10^−5^	3.5 × 10^−12^
CC5-6	7.4 × 10^4^	n.m.	n.m.
CC5-25	6.5 × 10^5^	2.9 × 10^−5^	4.4 × 10^−11^
CC5-12	1.1 × 10^6^	2.7 × 10^−4^	2.4 × 10^−10^
CC5-14	1.5 × 10^7^	1.0 × 10^−2^	7.4 × 10^−10^
CC5-20	2.4 × 10^6^	3.5 × 10^−3^	1.6 × 10^−9^

n.m. = no measurable dissociation was observed.

### Structure of CC5-17 with GP38 Hoti

The cross-clade CCHFV and cross-species (Aigai virus) binding potential of CC5-17 was explored using BLI. The same pattern as c13G8 was observed, in which CC5-17 bound to GP38 from CCHFV IbAr10200, Turkey-2004, and Hoti, but it did not engage with Aigai virus GP38 (Fig. 2e). Like the SPR results, little to no appreciable dissociation was observed between CC5-17 and the various GP38s (Fig. 2e, Table 1). Subsequently, a 3.8 Å structure of GP38-CC5-17 was determined to gain insights into how this high-affinity anti-GP38 mAb interfaced with the site I (PDBID 8DDK) (Fig. 2f, Supplementary Table 1). As with the mAb-c13G8 complex structure, the binding interface was well characterized with a comparable interface surface of 776.5Å^2^ (Supplementary Fig. 3b)^26^. At first glance, CC5-17 appears to bind globally to a similar area with three main determinants conferring binding. The first is LCDR3 which partially interacts with the N-terminus, and the second interacts with α-helix 2 of GP38. The third interaction is the HCDR3 which interacts with a larger hydrophobic pocket on top of GP38 and the large 20 AA flexible loop (Fig. 2g).

### Comparison of c13G8 and CC5-17 binding to GP38 Hoti

**Fig. 3:**
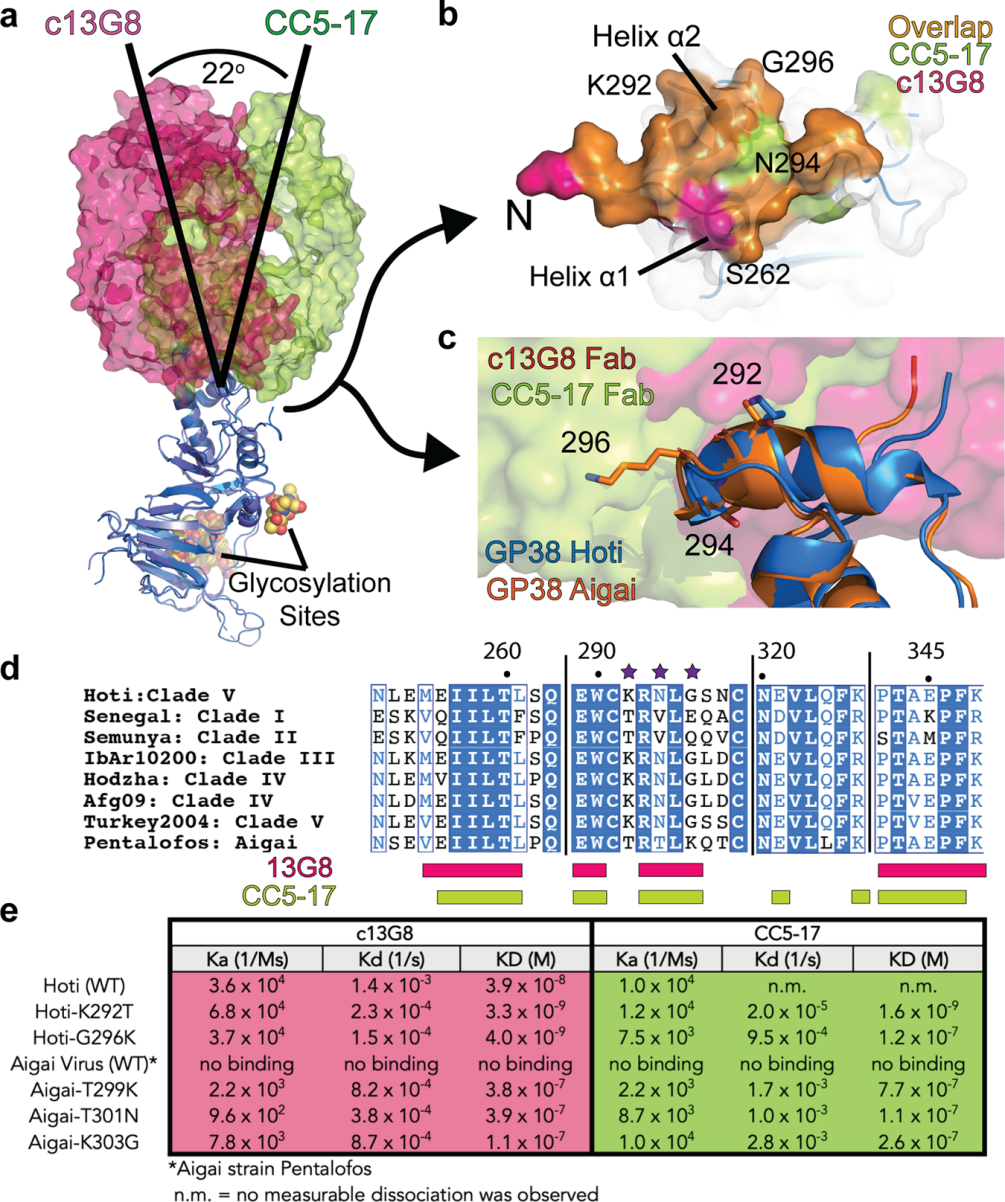
GP38 site I investigation. **a** Comparison of c13G8 (magenta) vs. CC5-17 (lime) engagement with GP38 site I epitope. **b** Surface area comparison of site I epitope, magenta showing surface area of 13G8 engagement, lime denoting surface area of CC5-17 engagement, and orange denoting the shared surface area of the two Fabs. **c** Homology model of GP38 from Aigai virus (orange) overlay on complex structure. **d** Sequence alignment of relevant residues on GP38, purple stars denote sites of interest. **e** Single mutations were conducted at GP38 Hoti sites 292 and 296 and Aigai virus GP38 sites 299, 301, and 303. These mutant proteins were then tested via BLI to obtain their binding kinetics.

A comparison of the interactions of c13G8 and CC5-17 with site I reveals a different angle of engagement with GP38. This is observed by the 22° difference between the core axis of the Fabs from c13G8 and CC5-17 (Fig. 3a). As previously mentioned, both antibodies rely on three areas of GP38 (residues 253–261, 289–294, and 343–349). The overall binding interface is conserved through the two antibodies with minor differences between them (Fig. 3b). The 22° shift enables CC5-17 to engage GP38 more thoroughly than that of c13G8. This includes CC5-17’s LCDR3 engaging with α-helix 2, several residues appear to interact at this site, including a salt bridge between GP38’s E289 and CC5-17’s LCDR3 R93, and the LCDR3 Y92 proximity to GP38 can engage either with the hydrophobic pocket or cause hydrogen bonding events with α-helix 2 (Supplementary Fig. 5a). In addition, CC5-17’s HCDR3 penetrates the hydrophobic pocket and causes a shift of GP38 residues 340–345, not previously seen in comparison to the c13G8-GP38 Hoti structure or that of unbound GP38 Hoti and IbAr10200 (Supplementary Fig. 5b).

### Origins of c13G8 and CC5-17 mAbs selectivity for CCHFV over the Aigai virus

To elucidate the molecular basis for this difference in affinity at site I for both c13G8 and CC5-17, the site I epitopes of these two mAbs were compared across CCHFV Clades I–V and Aigai Virus. When comparing the sequences of IbAr10200, Turkey-2004, Afg09, Hoti, and Aigai virus, there are several deviations between Aigai and the CCHFV strains that reside along α–helix 2 at residues 292, 294, and 296 (Fig. 3d). At site 292 a lysine is replaced by a threonine, while at site 294 an asparagine is now either a valine (CCHFV Clade I/II) or a threonine (Aigai virus). Site 296 has the most variation between the evaluated viruses. Instead of a glycine found in three of the five CCHFV clades (Clades III, IV, and V), it can be a glutamine (Clade I), a glutamic acid (Clade II), or a lysine (Aigai virus). To probe the impact of amino acid variation within the site I epitope, single amino acid mutations were introduced into Hoti GP38 to abolish binding. The reverse was also performed by introducing the single mutations into Aigai GP38 to re-establish binding. BLI was used to calculate binding affinities of 13G8 and CC5-17 against the new mutant GP38s to evaluate amino acid sites 292, 294, and 296.

The impact of GP38 Hoti containing single mutations drawn from Aigai virus on the affinity of c13G8 appears to be limited (Fig. 3e). The same was observed for the K292T mutant with CC5-17. As expected from the position of G296 within the GP38-CC5-17 interface, mutation to lysine had a significant impact on CC5-17 and its affinity. This impact lines up with the different binding angles between c13G8 and CC5-17. Specifically, amino acids with any side chain would create a steric clash with CC5-17, whereas such side chains could find the bulk solvent when bound to c13G8 (Fig. 3c). While many of the mutations in GP38 from the CCHFV Hoti strain had minimal impact in lowering c13G8 and CC5-17 affinity for GP38, adapting equivalent Aigai virus GP38 sites 299, 301 and 303 resulted in the restoration of measurable binding affinity (Fig. 3e). These trends suggest that the lack of c13G8 affinity for GP38 from the Aigai is largely due to a synergistic effect of multiple differences within site I. Altogether, this data also suggests more than one alteration within the site I at these amino acid positions would be necessary for c13G8 to lose significant activity, whereas having a glycine at position 296 is necessary for robust CC5-17 binding.

### Affinity of non-neutralizing antibodies to site I is not the sole driver of protective efficacy

**Fig. 4:**
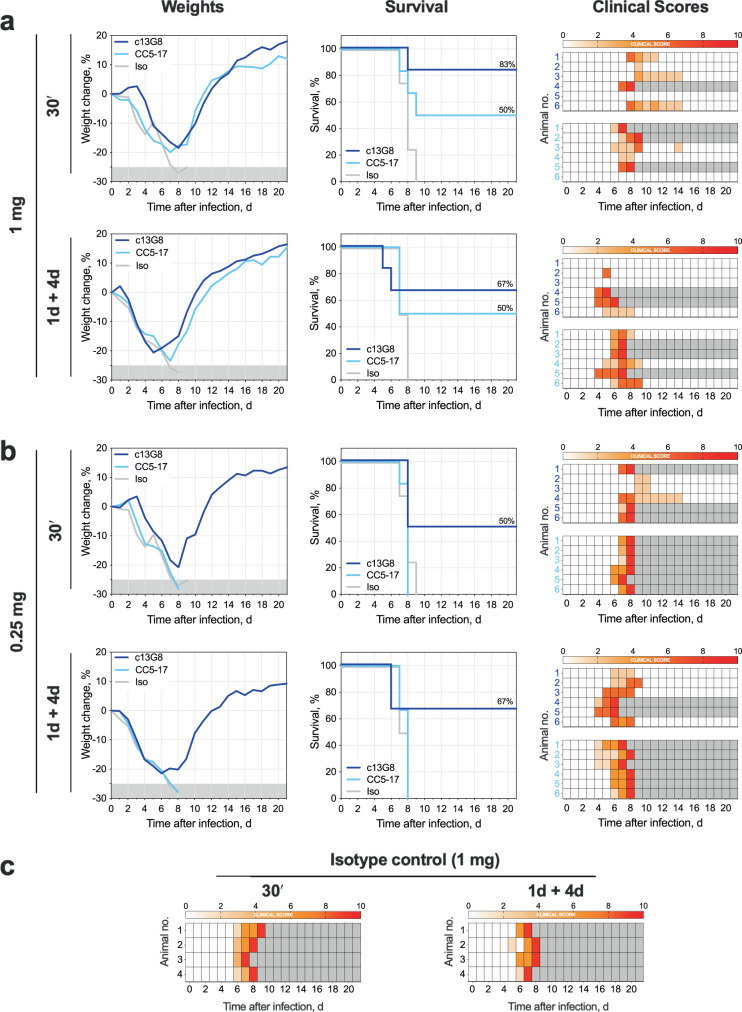
Efficacy of site I antibodies in mice against CCHFV infection. Groups of IFNAR^−/−^ mice were infected subcutaneously with CCHFV Turkey-2004 (target dose: 100 TCID50) and treated intraperitoneally with c13G8 (blue) or CC5-17 (light blue) (*n* = 6) at indicated timepoints and doses and followed for 21 days post-challenge. For this study, both antibodies were generated on the human IgG1 and kappa light chain backbone. Mean weight change (baseline at day 0), survival and clinical scores in mice following: **a** 1 mg mAb treatment at 30 min or +1/+4 days after challenge or **b**, 0.25 mg mAb dose treatment at 30 min or +1/+4 days after challenge. In parallel, groups of mice were treated intraperitoneally with IgG1 isotype control (1 mg dose only, *n* = 4) at indicated timepoints (mean weight loss and survival indicated by gray line). In contrast to mice treated with 1 mg of 13G8 or CC5-17, all mice treated with 1 mg of isotype control (at indicated timepoints, **c** developed severe clinical illness and succumbed to infection. Clinical scores ≥10 indicate end-point criteria. Gray boxes in clinical scores indicate animals removed from study due to fatal disease.

To investigate if increased binding affinity of CC5-17 confers higher protective efficacy than c13G8, we evaluated clinical course and outcome following treatment in the IFNAR^−/−^ mouse model of lethal disease. Groups of mice (*n* = 4–6 per group) were infected subcutaneously (SC) with a target dose of 100 TCID_50_ of CCHFV Turkey-2004 and subsequently treated intraperitoneally with standardized IgG1 site I mAbs or isotype IgG1 control. Two dosing regimens were evaluated (1 dose at 30 min post-infection or two doses at +1/+4 dpi) and two doses were tested for each regimen (1 mg or 0.25 mg). In our hands, efficacy of c13G8 treatment was comparable to previously published reports^9,11^. Dosing at 1 mg resulted in 66–83% survival and decreased with the lower dose of 0.25 mg (50–66% survival) (Fig. 4). Interestingly, despite higher binding affinity, CC5-17 conferred lower therapeutic efficacy than c13G8. Dosing at 1 mg resulted in 50% survival and all animals succumbed to infection by 7 dpi when the dose was reduced to 0.25 mg. These data further support the ability of c13G8 to provide protection after infection. The path to establish a more efficacious mAb through competition assays and binding affinity is complex, as seen here with CC5-17, in which antibodies targeting the same site can have drastically different efficacy, which raises new questions about the characteristics needed for anti-GP38 to provide protection. These data also indicate the importance of timing of mAb administration in treatment of CCHF, supporting early commencement of treatment as a priority; while both approaches (30 min or +1/+4 day) afforded comparable protection at the same dose, animals receiving their first treatment later had more acute onset of clinical signs as compared to those treated earlier in the infection course^9,11^.

## Discussion

Neutralizing antibodies have desirable therapeutic activity against some viruses, especially with recent work highlighting COVID-19^32–34^. The therapeutic role for non-neutralizing antibodies is becoming more evident for high-risk pathogens like Ebola, Lassa, and Nipah toward preventing severe disease^35–39^. Therapeutic development toward CCHFV has proven difficult, but data from GP38-based DNA vaccines and anti-GP38 mAb studies have demonstrated that GP38 is immunorelevant and a viable anti-viral target^11,16,17^. Here, we show a complex structure of GP38 with that of c13G8 and the human-derived mAb CC5-17.

Although the human-derived mAbs boasted a clear increase in affinity over its mouse-derived counterpart, c13G8, CC5-17 demonstrated lower efficacy in vivo. Previous research discussed that binding affinity could be a determinant of efficacy toward site I^11^. As seen structurally, the epitope between 13G8 and CC5-17 is largely the same. However, these two antibodies approach GP38 at two different angles, which contributes to their differences in binding affinity. Although CC5-17 has a tight interaction with GP38 which result in an unmeasurable dissociation rate, its Ka is up to tenfold lower than that of c13G8 (Table 1) which might have contributed to its lower efficacy. As a whole our studies suggest that site I mAbs can have drastically different efficacy and the drivers of protection apart from binding affinity need to be further investigated.

GP38’s function is still unknown, but recent evidence points to it localizing to the outside of viral particles and to the surface of cells. It is possible that the increase in association rate assists in clearance of not only virus but infected cells, or that the angle of approach bears more relevance on the cell surface or binding of GP38 in precursor or partially processed forms linked to the GPC MLD. The change in binding angles dictating the strength of effector function has been demonstrated in the case of antibodies to the HIV-1 envelope glycoprotein^40^. In the case of GP38 the angle of antibody binding could be critical for maximum complement binding, as previous studies show that 13G8 prevents viral spread to the liver, a direct elimination of the virus or virus infected cells could be a mechanism^11^. If not for the association rate, how GP38 localizes to the outside of these particles needs to be considered and if the orientation of the localized GP38 influences binding of mAbs. This would also need to be considered through site II–V mAbs and if they provide any efficacy.

The original study with the murine version of 13G8 indicated an important role of complement in efficacy^11^. 13G8 has been engineered a few ways but the protective efficacy has been similar between the murine and humanized versions, suggesting that the Fc domain functionality is not diminished between the two versions^17^. Another study suggested that Fc effector function was not critical, however that study utilized a 13G8 with a LALA-PG mutation on a human IgG1 backbone which is reported to impede Fc receptor binding but does not impair complement activity^41^. Therefore, complement activity may be necessary for maximum protection with 13G8, but future studies are required to fully understand the mechanism of protection and the role of complement.

Of interest structurally and biochemically is that both c13G8 and CC5-17 partially bind to the N-terminus near the furin cleavage site. Through processing of the CCHFV GPC, GP160 and GP85 are further cleaved by furin to yield the free GP38 and MLD^42^. The proximity of the site I antibodies epitope with furin cleavage suggests that inhibition of processing might contribute to mAb efficacy as this cleavage contributes to CCHFV infectivity. In addition, furin could cleave immature forms of Pre-Gn upon viral entry if associated with the virion, as observed for the surface glycoproteins of coronaviruses and flaviviruses^43,44^. If the efficacy of GP38 mAbs were limited to those binding the N-terminus capable of blocking furin processing, this would focus future mAbs development efforts toward this specific activity.

The isolation of human-derived antibodies like CC5-17 that provide partial protection in animals further solidifies the immunogenicity importance of GP38 and importance of antibody response to the site I. In addition, patient CC5 was observed to have a relatively low SHM rate in their antibody repertoire. High hypermutation rates are seen within chronic infections like Human Immunodeficiency Virus (HIV), where the SHM rates are 30–50% in comparison to SHM rates of 3–14% observed here with CCHFV, which produces an acute infection. This is promising as it appears that human immune systems can deliver protective antibodies against CCHFV with low SHM rates. Alongside if GP38 were to be used in future vaccinations, this can be done with relative ease as other vaccination efforts with multiple doses against viruses like HPV have SHM around 8–10%^45,46^. The ability of both mice and humans to generate antibodies that have substantial cross-protection potential by targeting a conserved site I on GP38 also bodes well for developing GP38-inspired antigens for vaccine platforms. This could take the form of chimeric GP38s, GP38 cocktails, or GP38 mini proteins^47,48^.

From a broad-spectrum therapeutic perspective, the structural insights gained via the GP38-c13G8 and GP38-CC5-17 structures suggest that anti-GP38 mAbs have considerable cross-protection potential. This may be of considerable benefit, given that neutralizing G_C_ mAbs appear to be limited in breadth of protection when it comes to the five phylogenetic clades of CCHFV M-segment. Recently, a bispecific mAb DVD-121-801 has shown robust post-exposure protection against IbAr10200^9^. While it was shown to have strong neutralization against the CCHFV laboratory isolate IbAr10200 and its Oman counterpart, weak neutralization was observed for Clade V Kosovo/Hoti and Turkey-2004. In contrast, c13G8 can protect post-infection against the major clinically relevant strain Turkey-2004. (Clade V), and past studies confirmed it can protect against IbAr10200 (Clade III)^11,17^. Through our structural and biochemical studies, we illustrate how site I antibodies bind across the major clinical strains of CCHFV (Clades III–V). Antibody development toward CCHFV also needs to target highly conserved antigenic sites, to prevent viral mutations from making the mAb obsolete with new variants. Through our studies, we have shown that several mutations need to occur in GP38’s α-helix 2 to cause the obsolescence of 13G8 and CC5-17 as therapeutic options. Also, these mutational studies highlight how polymorphisms found in CCHFV Clade I–II are unlikely to impact 13G8 binding but may for CC5-17.

Through our work we have established the structural and biochemical characteristics of GP38’s antigenic site I, with a mouse-derived anti-GP38 mAb and a human-derived anti-GP38 mAb. The characterization of this site provides a rationale for the development of vaccines and mAbs targeting GP38 site I, in addition, this site can be utilized for future vaccination methods to fully cover the whole spectrum of CCHFV genetic diversity (clades I–V) and potentially other nairoviruses.

## Disclaimer

Opinions, interpretations, conclusions, and recommendations are those of the author and not necessarily endorsed by the Centers for Disease Control and Prevention, the U.S. Army, or the Department of Defense.

## Methods

### Biosafety and ethics

All work with infectious virus or infected animals was conducted in a biosafety level 4 (BSL-4) laboratory at the Centers for Disease Control and Prevention (CDC). All animal experiments were approved by the CDC Institutional Animal Care and Use Committee (3102SPEMOUC) and performed in an AAALAC-approved facility.

### CCHFV GP38 production

Plasmid constructs to produce GP38 (Hoti, Aigai-Pentalofos, Afg-09, Turkey-2004, IbAr 10200) included the N-terminal region of CCHFV GPC from the N-terminal signal peptide to the end GP38 domain, excluding the C-terminal site-1 protease cleavage which was replaced with an HRV3C protease followed by an 8X HisTag and Twin-Strep-tag (GenBank: AWX63617.1, AVO00706.1, ADQ57289.1, ASW22359.1, AWX63620.1). The plasmid inserts were codon-optimized for expression in human cells and cloned into pTwistBeta vector. Expi293 cells (Thermo Fischer) were maintained in Expi293 Media (Thermo Fischer) based on their specifications. Expi293 were transfected with FectoPro reagent (Polyplus).

CCHFV GP38 expression plasmids were co-transfected with pCDNA3.1-human furin plasmid, generously provided by N.G. Seidah (Clinical Research Institute of Montreal), at a ratio of 4:1 in Expi293 cells. Four hours after transfection, kifunesine was added to a final concentration of 5 µM to inhibit the processing of complex glycans, which could interfere in the crystallization process. The cell supernatant was harvested between 5 and 6 days then the cells and debris were removed by centrifugation and filtered through a 0.2 µm filter. The sodium chloride concentration of media was adjusted to 500 mM final using 5 M NaCl solution. Supernatants were then run over a Histrap-Excel nickel column (Cytiva) and eluted using 500 mM Imidazole. The eluted proteins were digested overnight with HRV3C protease. The cleaved GP38 was purified by size exclusion chromatography on a Superdex 200 column.

### Patient recruitment

Patients infected with CCHFV who were admitted to Farabi Hospital (Karadeniz Technical University, Trabzon, Turkey) and in the convalescence phase were recruited. Blood was drawn and separated into PBMCs and Plasma. All patients signed informed consent, and the study was approved by IRBs in Karadeniz Technical University and the University of Maryland, Baltimore.

### Anti-GP38 antibody ELISA

The microtiter Immulon 2 HB 96-well flat bottom plates (Immuno Chemistry Technologies, Bloomington, MN) were coated with 0.2 µg of the GP38 antigen (Hoti strain)-overnight and blocked with 10% dried milk in TBS and 0.1% NP-40 (Blotto solution). For running ELISA, a 1:100 dilution for plasma samples and 10 µg/ml of the isolated mAbs were used as starting point. Following 1:2 serial dilutions of the samples, they were added to the wells and incubated for 1 h at 37 °C. After washing the plates for 4 times with TBS-T (Tris-buffered saline with 0.1% Tween® 20 detergent), a 1:1000 dilution of goat anti human IgG antibody conjugated to alkaline phosphatase (Southern Biotech, Birmingham, AL) was added to the wells and microplates were incubated for 1 h at 37 °C. The wells were washed 6 times with TBS-T and the substrate (BluePhos® Microwell, Seracare, Milford, MA) was added to each well for 15 min at 37 °C. Then the signal was read at 650 nm. The tests were done in duplicates, and the average of the background subtracted results are reported. To analyze the data, they were fitted using non-linear regression (curve fitting) using Prism 5 for Windows version 5.04.

### Isolation and generation of human anti-GP38 antibodies

The antibody was isolated from a donor CC5 (recruited as noted above). A combined transcriptomics and genomics approach was used for the identification of the specific antibodies. The circulating anti-GP38 antibodies in plasma were isolated using sequential affinity chromatography. The isolated antibodies were digested with proteases (trypsin, chymotrypsin, and Glu-C), and the resulting peptides were subjected to Mass spectrometry to construct a proteomics library (Northwestern Proteomics Center of Excellence, which was not involved in the data analysis). For the genomics analysis, the memory B cells were isolated from PBMC using EasySep™ Human Memory B Cell Isolation Kit (Cat#17864), then the single cell suspension was loaded onto the 10x Genomics Chromium Controller, microfluidics chip, and the VDJ library were prepared based on manufacturer’s instruction. The LC-MS/MS peptide spectra were searched against the memory B cell database using PEAKS Studio (v 7.5) (Bioinformatics Solutions Inc., Ontario, CA). The sequence of the matched paired heavy and light chains was used for the production of the mAbs. For this purpose, the variable regions of the antibodies were cloned in the plasmids contained constant regions of the IgG1 or IgG3 and k or λ. The paired heavy and light chain plasmids were co-transfected into the FreeStyle-293 cells, and recombinant antibodies were purified from culture supernatants by Protein A affinity chromatography.

### Anti-GP38 Fab production

To produce His-tagged Fabs, Expi293 cells were transfected with 1:1 ratio of His-tagged heavy chain plasmid to untagged light chain plasmid. Fab containing supernatant were equilibrated and purified by immobilized metal affinity chromatography as previously described above for GP38 purification.

### Anti-GP38 mAb production

Expi293 cells were transfected with 1:1 ratio of heavy chain plasmid to light chain plasmid. The cell supernatant was harvested between 5 and 6 days then the cells and debris were removed by centrifugation and filtered through a 0.2 µm filter. The mAb supernatant was equilibrated to a pH of 7.4, 200 mM NaCl, and 40 mM sodium phosphate dibasic and then flowed over a mAb Select Sure column (Cytva). Bound protein was eluted via 100% elution buffer (100 mM sodium Citrate pH 3.4) and 1 ml fractions were neutralized using 200 mM of Tris-Base pH 10.0. Fractions were confirmed using SDS-Page and then dialyzed to 200 mM NaCl, 4 mM Tris-HCl pH 8.0 for use in BLI studies.

### Crystallization of GP38-Fab complexes

Proteins were equilibrated to 4 mM Tris-HCl pH 8.0, 200 mM NaCl, a 1:1.2 ratio of GP38 to Fab was incubated for 4 h. After incubation, the complex was purified over a Superdex 200 column using a Biorad NGC. Fractions were collected and run using SDS-PAGE to confirm purity. Fractions containing the complex were then concentrated to 10–13 mg/ml using a 10 K Vivaspin Centrifugal Concentrator. Nextel crystal screens solutions were plated via an SPT Lab Tech Mosquito using a 1:1 ratio of protein to well solution hanging drop to a total volume of 600 nL. Drops were checked over 2–3 weeks for crystal formation. GP38 Hoti formed in a solution of 20% PEG3350, 0.4 M NH_4_I. 13G8Fab-GP38Hoti was formed in a solution of 1.1 M sodium malonate, 0.1 M HEPES pH 7.0, and 0.5% Jeffamine ED-2001, while CC5-17 Fab-GP38Hoti was formed in a solution of 20% PEG1000, and 0.1 M HEPES pH 6.4. Crystals were then flash-frozen in liquid nitrogen.

### Data collection, processing and refinement

Data were collected at the Advanced Photon Source (APS) beamlines 19-BM. Data Processing was performed using HKL-2000 (v 719.2) and CCP4 suite (v 8.0.005). Molecular Replacement was performed using Phaser-MR (simple one-component interface) for 8DC5 and Phaser-MR (full-featured) for 8DCY and 8DDK out of the Phenix suite of programs. To phase the complexes, 13G8 Fab and CC5-17 Fab models were generated using SWISS-MODEL, while the GP38-Hoti model was generated using MODELLER (v 9.22) (PDBID 6VKF)^49–54^. After phasing structures went through multiple rounds of refinement in Coot (v 0.9.8.3) and Phenix (v1.20.1)^55,56^.

### BLI kinetics assay mAb to GP38

An Octet R8 BLI protein analysis system (Sartorius) was used to determine binding kinetics of GP38 mAb. Anti-hIgG Fc Capture (AHC) sensors from Sartorius which was dipped into baseline 1× Octet kinetics buffer for 60 s. Sensors then encountered a 1 µg/mL antibody solution of either c13G8 or CC5-17 for 150 s as a loading step, following loading, they were then dipped into a second baseline containing 1× Octet Kinetics buffer. Sensors were then dipped into varying nM concentrations (0.3125, 0.625, 1.25, 2.5, 5.0, 10.0, 20.0) of either CCHFV strains IbAr10200, Turkey-2004, and Hoti, or Aigai virus strain Pentalofos GP38 for 300 s as an association step, and then transferred back to 1× Octet kinetics buffer for 600 s as a dissociation step. KD were determined from BLI curves obtained from the analysis of GP38 concentration ranging from 0.3125 to 20 nM. Data from binding kinetics curves were fit globally using a 1:1 binding model with Sartorius Analysis software (v 12.2).

### BLI mutational studies

Protein A (ProA) BLI sensors from Gator Bio were dipped into baseline Gator Bio Kinetics Buffer (K Buffer) for 120 s. Sensors then encountered a 25 µg/mL antibody solution of either c13G8 or CC5-17 for 300 s as a loading step, following loading they were then dipped into a second baseline containing Gator Bio K Buffer. Sensors were then dipped into varying nM concentrations (1500, 500, 166.67 or 250, 50, 10) of CCHFV strain Hoti, Aigai strain Pentalofos, or mutants of these for 1000 s as an association step, and then transferred back to Gator Bio K Buffer for 4000 s as a dissociation step. KD’s were calculated via Gator Bio’s software (Gator Part 11 v 2.7).

### Surface plasmon resonance

On and off rate and affinity measurements were performed on Biacore X100. Each antibody (CC5’s mAbs and c13G8) was immobilized onto Protein A chip (Biacore Series S Sensor Chip) at an Rmax of ~50 RU (response units). Measurements were made using serial dilutions of the recombinant GP38 protein, CCHFV Hoti GP38, starting from 20 to 0.625 nM in HBS-EP + buffer (0.1 M HEPES, 1.5 M NaCl and 0.5% v/v Surfactant P20, pH 7.4). The protein A chip was regenerated by 10 mM Glycine-HCl, pH 1.5. The data were analyzed with the Biacore X100 evaluation software (v 2.0.4) with a 1:1 binding model.

### BLI competition assay

Site I, II, and III antibodies were bought from BEI resources. BLI competition was performed through Sartorius Octet Discovery software (v 12.2). PentaHis Sartorius probes were soaked into a solution of 10 µg/ml Turkey2004 GP38-his/strep for 60 s. Following loading of the probe these were then dipped into Sartorius 1× kinetics buffer for 30 s. Probes were then dipped into the first antibody solution 10 µg/ml for 600 s to saturate. Probes were then dipped into kinetics buffer for 30 s and then dipped into competing a 10 µg/ml antibody solution for 300 s. Following competition, the probes were then regenerated in 10 mM glycine for 10 s and then dipped into kinetics buffer to wash the probes for 10 s. This step was done three times to fully dissociate the previous proteins. Processing was performed by taken by the maximum signal during each step involving the antibody. Calculation of percent inhibition was performed by taking the competing antibodies signal and dividing by the max signal by the same antibody during when it was used as a saturating step. This number was multiplied by a 100 to give a percentage inhibition, and lower percentages indicate less shift in nm and more competition between two antibodies and calculations were performed in Microsoft Excel.

### Neutralization of rCCHFV/ZSG by the monoclonal antibodies

Threefold, 9-point dilution series of the monoclonal antibodies were prepared in DMEM supplemented with 2% heat-inactivated FBS, sodium pyruvate and penicillin-streptomycin (Gibco). These dilutions as well as no-antibody controls, were mixed 1:1 with rCCHFV/ZSG (strain IbAr10200)^57^ grown on Huh7 cells diluted in the same so that the final antibody concentrations ranged from 10 µg/mL to 1.52 ng/mL and that virus titer of appr. 150 focus-forming units/40 µL was achieved. The mixes were incubated for 1 h at +37 C. Then, medium was removed from Vero-E6 cells growing in 96-wells, and quadruplicate wells were infected with 40 µl each at +37 °C, 5% CO2 with periodic shaking. After 1 h, the inocula were removed and the cells overlaid with MEM (Gibco) containing 1.25% carboxymethylcellulose (Sigma-Aldrich), 4% HI-FBS, sodium pyruvate, and penicillin-streptomycin. Fluorescent foci were imaged 2 days later using a Cytation3 instrument (BioTek), and the focus counts normalized against no-antibody controls. To graph the data and to determine the FRNT50 value, a 4-parameter non-linear regression analysis with top constraint of 1 was performed with GraphPad Prism v9.0 using log-transformed antibody concentrations.

### Generation of c13G8 and CC5-17 for animal studies

Plasmids of c13G8 and CC5-17 were standardized to a human IgG1 Fc backbone and were then expressed by transient transfection of expiCHO-S cells. Briefly, expiCHO-S cells were transfected with plasmids of the heavy and light chains of antibodies and expressed according to the manufacturer’s instructions for 10 days at 32 °C. Culture supernatants were collected and clarified, and antibodies were purified with HiTrap MabSelect Sure pcc (Cytiva) protein A affinity chromatography column on AKTA start fast protein liquid chromatography system. The CC5-17 antibody was eluted with pH2.2 glycine and c13G8 was eluted using IgG Elution Buffer (Thermo Scientific). Eluted fractions were neutralized with 2 M Tris. Following neutralization fractions containing antibodies were applied to HiLoad 26/600 Superdex 200 pg column to both improve the purity and buffer exchange to PBS. Pure fractions of antibodies were pooled, quantified by Nanodrop 2000c (Thermo Scientific), and stored at −20 °C. For the animal experiments, aliquots of mAbs were diluted in sterile PBS pH 7.4 at 0.25 mg/ml and 1 mg/ml for c13G8 and CC5-17 and 1 mg/ml for the isotype control. Diluted antibodies were further quantified using BLI in Octet R8 (Sartorius) with Protein A biosensors to confirm accuracy of the doses.

### Evaluating c13G8 and CC5-17 treatment against CCHFV in mice

B6.129S2-*Ifnar1*^*tm1Agt*^/Mmjax mice (MMRRC Stock No: 32045-JAX; male and female, 5–6 weeks old) were housed in a climate-controlled laboratory (68–79 °C and 30–70% humidity) with a 12 h day/night cycle; provided sterilized commercially available mouse chow (Laboratory Autoclavable Rodent Diet 5010, LabDiet), and sterile water *ad libitum*; and group-housed on autoclaved bedding (Bed-o’Cobs® ¼”, Anderson Lab Bedding; Care Fresh, Healthy Pet; Enviro-Dry, Shepherd Specialty Papers) with cotton nestlets in an isolator-caging system (Tecniplast GM500, West Chester, PA, USA) with a HEPA-filtered inlet and exhaust air supply. Mice were infected SC in the interscapular region with 100 µL of CCHFV Turkey-200406546 (passaged 1× suckling mouse brain and 1× in SW13 cells; GenBank: KY362517, KY362519, KY362515) under isoflurane anesthesia (target dose: 100 TCID_50_; back-titer dose: 63 TCID_50_). The virus stock was verified by next-generation sequencing and confirmed mycoplasma free. Viral stock titers and inoculum back-titers (calculated as TCID_50_) were determined by a method based on that of Reed and Muench on BSR-T7/5 cells fixed and stained at 5 dpi (rabbit anti-CCHFV NP pAb, IBT Bioservices 04-0011; 1:2500) and Alexa-488 goat anti-rabbit secondary antibody (IgG H&L cross-adsorbed, Invitrogen A11034; 1:2500). Groups of 6 mice (3 female and 3 male) were treated intraperitoneally (IP; 500 µL total volume) with monoclonal antibody (13G8 or CC5-17) 30 min post-challenge or at both 1- and 4-days post-challenge with 0.25 or 1.0 mg of antibody. Groups of 4 mice (2 female and 2 male) were mock treated IP with IgG1 isotype control (1 mg dose). Baseline weights were obtained at day 0, prior to inoculation, and mice were monitored daily for 21 dpi. Clinical signs in mice were scored based on 14 parameters: 2 points each for quiet, dull, responsive (QDR) disposition, hunched back or ruffled coat; 3 points each for dehydration or abnormal huddling/hypoactivity; 5 points each for ataxia/circling/tremors/paresis, abnormal breathing, or anemia; 7 points for weight loss of >20%; 10 points each for inability to bear weight, paralysis, frank hemorrhage or bleeding, moribund state, or weight loss of >25%. Animals were humanely euthanized when end-point criteria were reached (clinical score ≥ 10) or at study completion (21 dpi). Mean clinical scores were calculated by dividing the daily sum of all scores in an experimental group by the total by the number of animals remaining.

### Reporting summary

Further information on research design is available in the Nature Portfolio Reporting Summary linked to this article.

## Supplementary information

Supplementary Information

Reporting Summary

## Data Availability

All data generated or analyzed during this study are included in this published paper, source data file and supplementary files. Source data are provided with this paper. Data that support the findings of this study are also available from the corresponding author upon request. Atomic coordinates and structure factors have been deposited in the Protein Data Bank with PDB IDs 8DC5, 8DCY, and 8DDK. Source data are provided with this paper.

## Source data

Source Data
Peer Review File
