# Supplementary material for: Structural characterization of protective non-neutralizing antibodies targeting Crimean-Congo hemorrhagic fever virus
Source: Nat Commun. 2022 Nov 26;13:7298. doi: 10.1038/s41467-022-34923-0 (PMC9701186; doi:10.1038/s41467-022-34923-0)
Supplement: Supplementary file 1 — Supplementary Information [file 41467_2022_34923_MOESM1_ESM.pdf]

Supplementary Information

Structural Characterization of Protective Non-Neutralizing Antibodies targeting Crimean-Congo Hemorrhagic Fever Virus

Authors: Ian A. Durie[1#], Zahra R. Tehrani[2#], Elif Karaaslan[3,4], Teresa E. Sorvillo[3], Jack McGuire[4], Joseph W. Golden[5], Stephen R. Welch[3], Markus H. Kainulainen[3], Jessica R. Harmon[3], Jarrod J. Mousa [6,7], David Gonzalez[4], Suzanne Enos[1], Iftihar Koksal[8], Gurdal Yilmaz[9], Hanife Nur Karakoc[10], Sanaz Hamidi[9], Cansu Albay[9], Jessica R. Spengler[3], Christina F. Spiropoulou[3], Aura R. Garrison[5], Mohammad M. Sajadi[2], Éric Bergeron[1,3*], Scott D. Pegan[4,11*]

Affiliations:

[1]*Department of Pharmaceutical and Biomedical Sciences, College of Pharmacy, University of Georgia Athens, GA, USA*

[2]*Division of Clinical Care and Research, Institute of Human Virology, University of Maryland School of Medicine, Baltimore, Maryland, USA*

[3]*Viral Special Pathogens Branch, Division of High-Consequence Pathogens and Pathology, Centers for Disease Control and Prevention, Atlanta, GA, USA*

[4]*Division of Biomedical Sciences, University of California Riverside, Riverside, California, USA*

[5]*Virology Division, United States Army Medical Research Institute of Infectious Diseases, Fort Detrick, MD, USA*

[6] *Department of Infectious Diseases, University of Georgia College of Veterinary Medicine, Athens, GA 30602, USA*

[7]*Center for Vaccines and Immunology, University of Georgia College of Veterinary Medicine, Athens, GA 30602, USA*

[8] *Department of Infectious Disease and Clinical Microbiology, Acibadem University Atakent Hospital, Istanbul Turkey*

[9] *Department of Infectious Diseases, Karadeniz Technical University School of Medicine, Trabzon, Turkey*

[10]*Department of Infectious Disease and Clinical Microbiology, Bitlis State Hospital, Turkey*

[11]*Department of Chemistry & Life Science, United States Military Academy, West Point, NY, United States*

#Authors contributed equally to this work

*ebergeron@cdc.gov, *scott.pegan@medsch.ucr.edu

Supplementary Methods

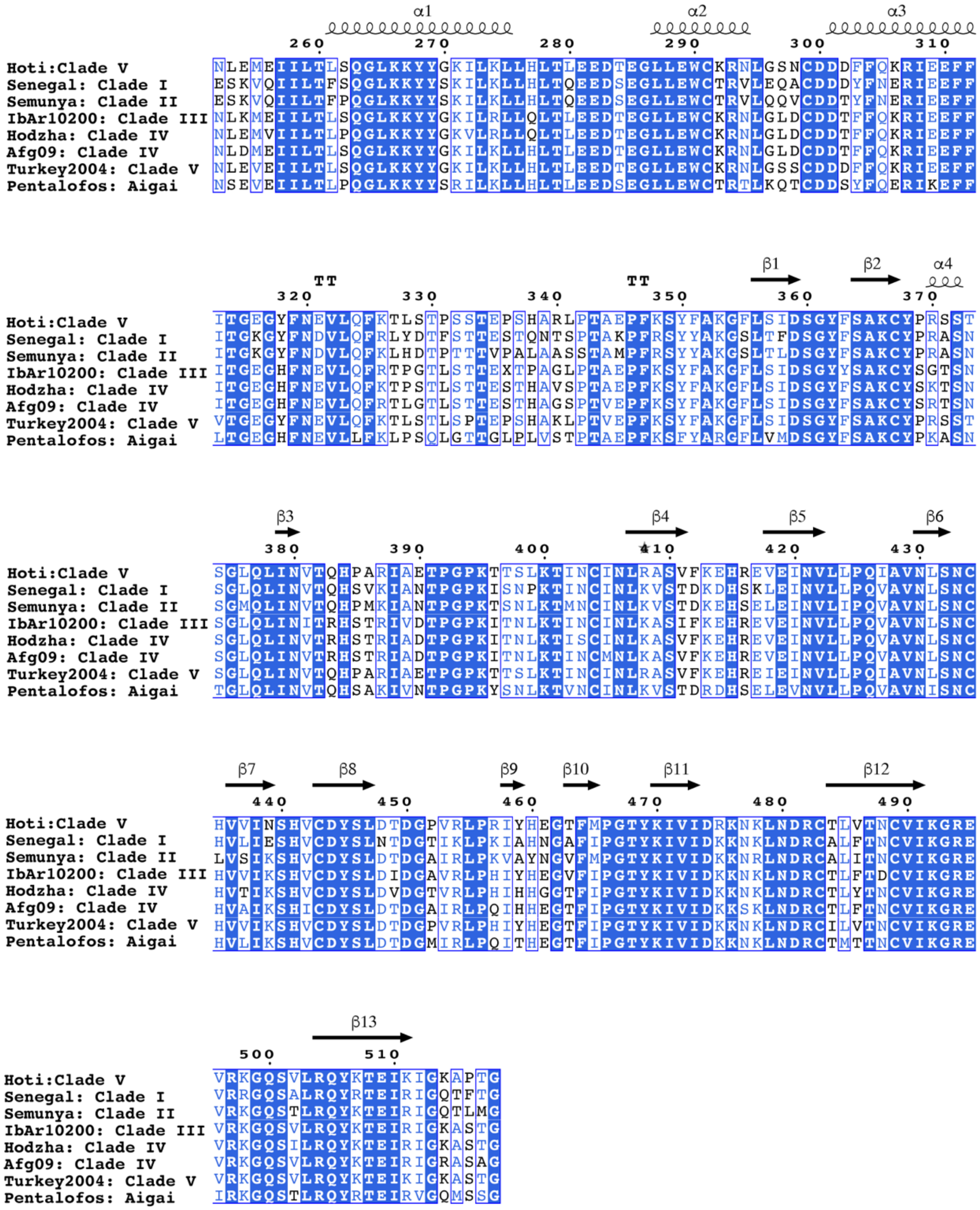

Supplementary Fig. 1 Sequence alignment of nairovirus GP38. CCHFV: Hoti (Accession number AWX63617.1), Senegal (Accession number ABB30027.1), Semunya (Accession number AAZ94860.1), IbAr 10200 (Accession number AWX63620.1), Hodzha (Accession number AAP29978.1), Afg-09 (Accession number ADQ57289.1) Turkey2004 (Accession number ASW22359.1), and Aigai Virus: Pentalofos (Accession number AVO00706.1). Similarity and alignment calculations were performed using Clustal Omega[47,48]. General outline and graphic was generated using EsPript[49]. Residue positions of high conservation are highlighted in Blue.

Supplementary Table 1: Data collection and refinement statistics

	**GP38 Hoti**	**GP38 Hoti complex with 13G8 Fab**	**GP38 Hoti complex with CC5-17 Fab**
**PDB**	8DC5	8DCY	8DDK
**Data collection**			
Space group	C_{121}	$P4_32_12$	$P4_32_12$
Wavelength (Å)	1.0	1.0	1.0
Cell dimensions			
a, *b*, *c* (Å)	171.63 75.32 62.21	175.92 175.92 93.22	175.67 175.67 92.16
a, *b*, g (°)	90 110.411 90	90 90 90	90 90 90
Resolution (Å)	50.00-3.2 (3.26-3.2)	50.0-3.60 (3.66-3.60)	50-3.85 (3.92-3.85)
$R_{pim}(\%)$	7.2 (40.8)	5.2 (55.4)	10.0 (47.9)
R_{merge}(%)	13.4 (79.4)	17.2 (182.9)	25.5 (128.6)
$cc_{1/2}$	0.98 (0.76)	1.00 (0.78)	0.88 (0.763)
I/s(*I*)	17.4 (3.6)	13.8 (8.7)	21.8 (1.0)
Completeness (%)	96.53 (93.34)	98.5 (92.1)	99.3 (98.7)
Redundancy	3.5 (3.8)	10.8 (10.2)	7.8 (6.6)
**Refinement**			
Resolution (Å)	29.2-3.2 (3.26-3.2)	45.1-3.62 (3.75 - 3.62)	40.81-3.86 (3.99 - 3.86)
No. reflections	11885 (1136)	17162 (1674)	13706(1303)
$R_{work}(\%)/R_{free}(\%)$	28.8/30.5	28.1/29.9	30.5/33.5
No. atoms	3642	5109	4668
Protein	3536	5050	4545
Ligand/ion	61	56	122
Water	45	3	1
B-factors	114	184	247
Protein	114	184	246
Ligand/ion	138	173	287
Water	87	143	232
R.m.s deviations			
Bond lengths (Å)	0.004	0.006	.004
Bond angles (º)	0.72	1.42	0.76
Estimate of Coordinate Error	0.44	0.58	0.45

*Highest resolution shell is shown in parenthesis.

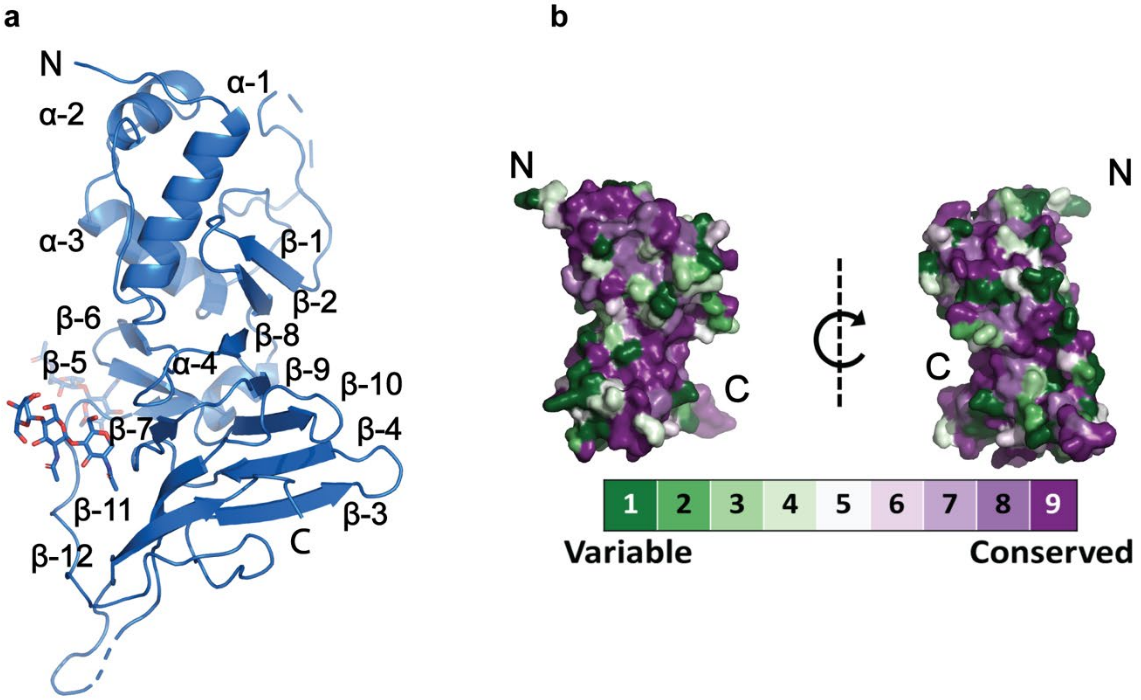

Supplementary Fig. 2 GP38 Diversity: **a,** Secondary Structure labeling of GP38 Hoti **b,** Consurf Analysis of GP38

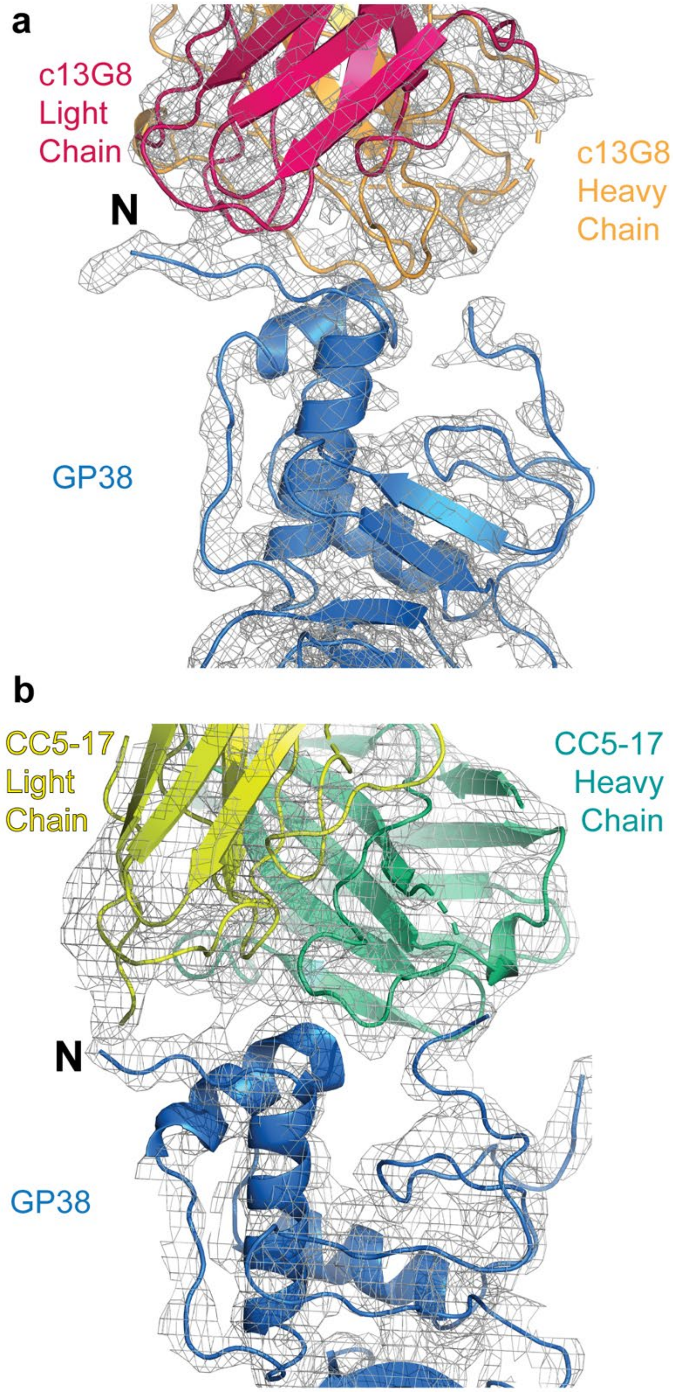

Supplementary Fig. 3 2Fo-Fc electron density of Fab-GP38 complex interaction a. 13G8-GP38 Hoti paratope (Top) and b. CC5-17-GP38 Hoti (bottom). The 2Fo-Fc electron density map is shown in grey.

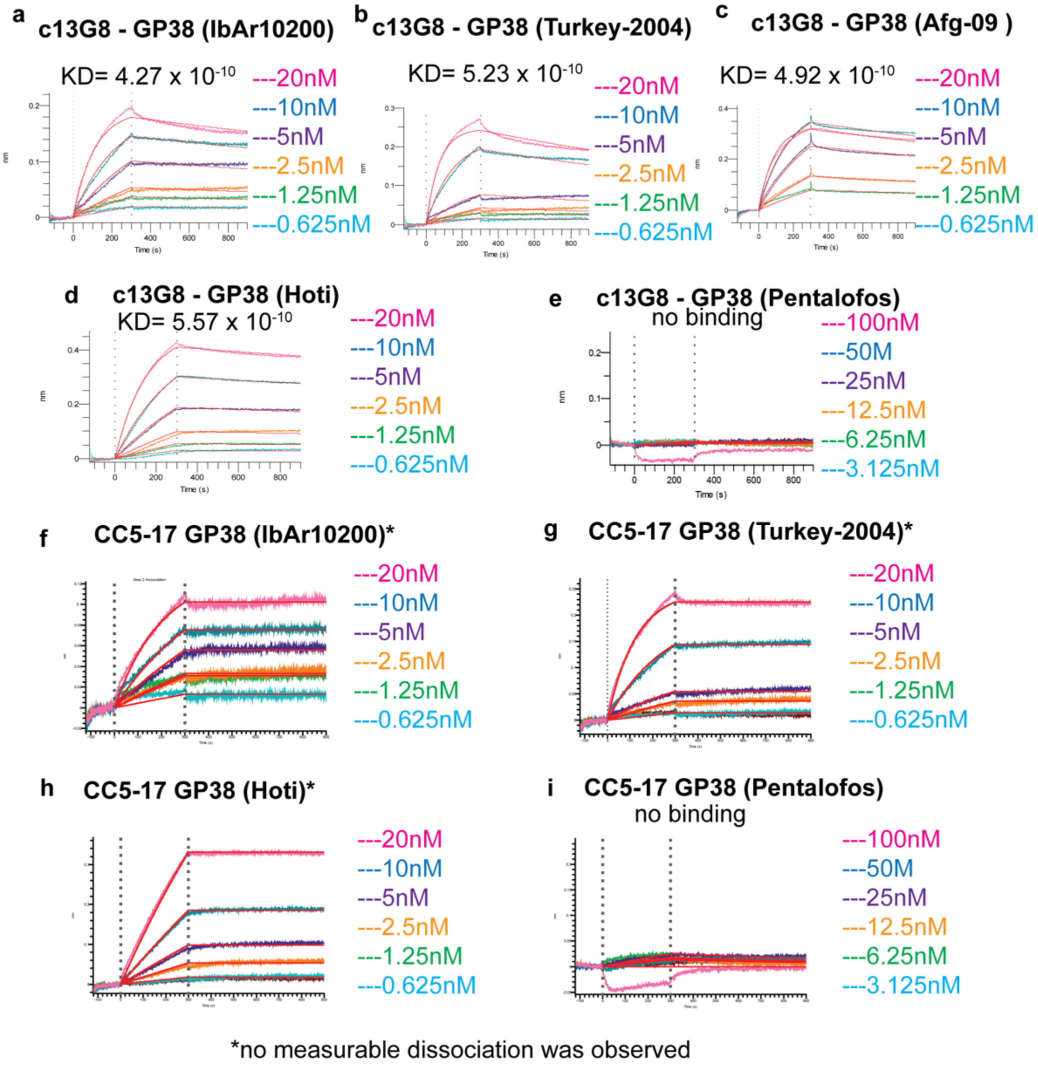

Supplementary Fig. 4 BLI curves of c13G8 and CC5-17 binding to nairovirus GP38. a, c13G8 to IbAr10200 GP38 b, c13G8 to Turkey-2004 GP38 **c**, c13G8 to Afg-09 GP38 **d**, c13G8 to Hoti GP38 **e**, c13G8 to Aigai strain Pentalofos **f**, CC5-17 to IbAr10200 GP38 **g**, CC5-17 to Turkey-2004 GP38 **h**, CC5-17 to Hoti GP38 **i**, CC5-17 to Aigai strain Pentalofos GP38

Supplementary Table 2. Antibody gene usage for non-neutralizing anti-GP38 mAbs

mAb	Gene family V	Gene family D	Gene family J	CDR3 Length	%SHM
CC5-17	IGHV5-51*01	IGHD3-10*01	IGHJ4*02	23	3%
	IGKV1-39*01	-	IGKJ1*01	7	9%
CC5-16	IGHV4-31*03	IGHD3-10*01	IGHJ4*02	15	13%
	IGLV2-11*01	-	IGLJ3*02	10	12%
CC5-6	IGHV4-31*03	IGHD3-3*01	IGHJ6*02	14	14%
	IGKV1-5*03	-	IGKJ1*01	8	7%
CC5-25	IGHV3-33*01	IGHD3-9*01	IGHJ6*02	15	5%
	IGLV3-1*01	-	IGLJ3*02	11	7%
CC5-12	IGHV4-34*01	IGHD6-25*01	IGHJ4*02	10	0%
	IGLV1-47*01	-	IGLJ1*01	12	5%
CC5-14	IGHV1-69*17	IGHD3-22*01	IGHJ5*02	20	14%
	IGLV1-40*01	-	IGLJ2*01	11	3%
CC5-20	IGHV1-69D*01	N/A	IGHJ6*02	15	7%
	IGKV3-20*01	-	IGKJ1*01	9	5%

*The gene family data was extracted from IgBLAST (https://www.ncbi.nlm.nih.gov/igblast/

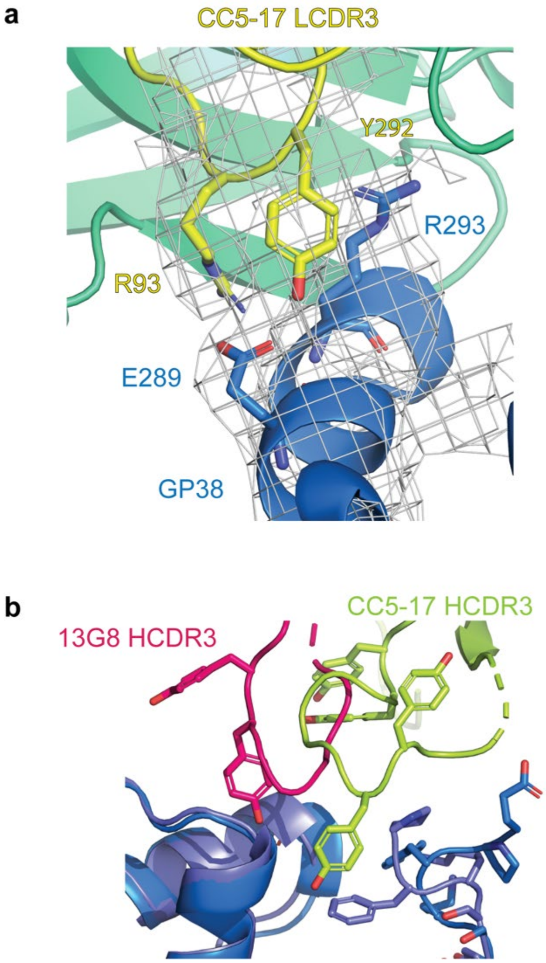

Supplementary Fig. 5 Comparison of c13G8 or CC5-17 interactions with Site I on GP38. **a,** Interaction of the CC5-17 LCDR3 on helix 2 of GP38 Hoti. **b,** Overlay of the two structures and dominance of their HCDR3,
