# Supplementary material for: Structural characterization of protective non-neutralizing antibodies targeting Crimean-Congo hemorrhagic fever virus
Source: Nat Commun. 2022 Nov 26;13:7298. doi: 10.1038/s41467-022-34923-0 (PMC9701186; doi:10.1038/s41467-022-34923-0)
Supplement: Supplementary file 2 — Reporting Summary [file 41467_2022_34923_MOESM2_ESM.pdf]

nature portfolio

Corresponding author(s): Drs. Scott Pegan and Eric Bergeron

Last updated by author(s): 2022-October-25

Reporting Summary

Nature Portfolio wishes to improve the reproducibility of the work that we publish. This form provides structure for consistency and transparency in reporting. For further information on Nature Portfolio policies, see our Editorial Policies and the Editorial Policy Checklist.

Statistics

For all statistical analyses, confirm that the following items are present in the figure legend, table legend, main text, or Methods section.

n/a	Confirmed	
☐	☒	The exact sample size (*n*) for each experimental group/condition, given as a discrete number and unit of measurement
☐	☒	A statement on whether measurements were taken from distinct samples or whether the same sample was measured repeatedly
☒	☐	The statistical test(s) used AND whether they are one- or two-sided<br>*Only common tests should be described solely by name; describe more complex techniques in the Methods section.*
☒	☐	A description of all covariates tested
☒	☐	A description of any assumptions or corrections, such as tests of normality and adjustment for multiple comparisons
☐	☒	A full description of the statistical parameters including central tendency (e.g. means) or other basic estimates (e.g. regression coefficient) AND variation (e.g. standard deviation) or associated estimates of uncertainty (e.g. confidence intervals)
☒	☐	For null hypothesis testing, the test statistic (e.g. *F*, *t*, *r*) with confidence intervals, effect sizes, degrees of freedom and *P* value noted<br>*Give P values as exact values whenever suitable.*
☒	☐	For Bayesian analysis, information on the choice of priors and Markov chain Monte Carlo settings
☒	☐	For hierarchical and complex designs, identification of the appropriate level for tests and full reporting of outcomes
☒	☐	Estimates of effect sizes (e.g. Cohen's *d*, Pearson's *r*), indicating how they were calculated

Our web collection on statistics for biologists contains articles on many of the points above.

Software and code

Policy information about availability of computer code

Data collection	BLI: Octet Discovery (v 12.2), Gator Part 11 (v 2.7); Crystallography: HKL-2000 (v 719.2), CCP4 suite (v 8.0.005), Phaser-MR (simple one-component interface) and Phaser-MR (full-featured) from Phenix (v1.20.1), Coot (v0.9.8.3); SPR: Biacore X-100 (v 2.0.4), Peaks Studio (v 7.5)
Data analysis	BLI: Microsoft Office, Octet Discovery (v 12.2), Gator Part 11 (v 2.7); Crystallography: HKL-2000 (v 719.2), CCP4 suite (v 8.0.005), Phaser-MR (simple one-component interface) and Phaser-MR (full-featured) from Phenix (v1.20.1), Coot (v0.9.8.3); SPR: Biacore X-100 (v 2.0.4), Peaks Studio (v 7.5)

For manuscripts utilizing custom algorithms or software that are central to the research but not yet described in published literature, software must be made available to editors and reviewers. We strongly encourage code deposition in a community repository (e.g. GitHub). See the Nature Portfolio guidelines for submitting code & software for further information.

Data

Policy information about availability of data

All manuscripts must include a data availability statement. This statement should provide the following information, where applicable:

- Accession codes, unique identifiers, or web links for publicly available datasets
- A description of any restrictions on data availability
- For clinical datasets or third party data, please ensure that the statement adheres to our policy

"All data generated or analyzed during this study are included in this published article, source data file and supplementary files. Source data are provided with this

paper. Data that support the findings of this study are also available from the corresponding author upon reasonable request. Atomic coordinates and structure factors have been deposited in the Protein Data Bank with PDB IDs 8DC5, 8DCY, and 8DDK."

Human research participants

Policy information about studies involving human research participants and Sex and Gender in Research.

Reporting on sex and gender	Monoclonal antibodies are derived from from adult donors >18 years of age, and demographic details were not collected. (not a population based study)
Population characteristics	All samples collected from adult donors >18 years of age, and demographic details were not collected. (not a population based study)
Recruitment	Patients infected with CCHFV who were admitted to Farabi Hospital (Karadeniz Technical 401 University, Trabzon, Turkey) and in the convalescence phase were recruited (invitations to join the study were done randomly).
Ethics oversight	All patients signed informed consent and the study was approved by the IRBs in Karadeniz Technical University and University of Maryland Baltimore

Note that full information on the approval of the study protocol must also be provided in the manuscript.

Field-specific reporting

Please select the one below that is the best fit for your research. If you are not sure, read the appropriate sections before making your selection.

☒ Life sciences ☐ Behavioural & social sciences ☐ Ecological, evolutionary & environmental sciences

For a reference copy of the document with all sections, see nature.com/documents/nr-reporting-summary-flat.pdf

Life sciences study design

All studies must disclose on these points even when the disclosure is negative.

Sample size	For mouse studies, samples sizes were determined based on consultation with a statistician and subsequently detailed for approval on IACUC protocol submission. As studies were exploratory (i.e., effect size and standard deviation were not known for power analysis), groups sizes were informed based on studies previously conducted in the model and reported (listed below), and confirmed to be within acceptable range of degrees of freedom (DF) using the 'resource equation' approach.<br>1. Spengler JR, Welch SR, Scholte FEM, Coleman-McCray JD, Harmon JR, Nichol ST, Bergeron É, Spiropoulou CF. Heterologous protection against Crimean-Congo hemorrhagic fever in mice after a single dose of replicon particle vaccine. Antiviral Res. 2019 Oct;170:104573. doi: 10.1016/j.antiviral.2019.104573. Epub 2019 Aug 1. PMID: 31377243; PMCID: PMC6773275.<br>2. Spengler JR, Welch SR, Scholte FEM, Rodriguez SE, Harmon JR, Coleman-McCray JD, Nichol ST, Montgomery JM, Bergeron É, Spiropoulou CF. Viral replicon particles protect IFNAR-/- mice against lethal Crimean-Congo hemorrhagic fever virus challenge three days after vaccination. Antiviral Res. 2021 Jul;191:105090. doi: 10.1016/j.antiviral.2021.105090. Epub 2021 May 24. PMID: 34044061; PMCID: PMC9250103.
Data exclusions	No data were excluded.
Replication	All attempts at replication were successful, if any measures were not replicated they are noted below:<br>Data from mouse studies (ifnar ko mice) included weights, clinical scoring, and clinical outcome (lethal/survivor). Replicate data acquisition was achieved overall by experimental group sizes. Replicate weight readings were obtained for individuals only when indicated (e.g., weight gain or loss exceeded expected range, or was not consistent with presence or absence of concurrent clinical signs). Clinical scoring of individual mice was repeated daily and determined by two independent assessors using a predetermined scoring system with objective and subjective measures. Replicates were not obtained for outcome data due to the nature of the parameter.
Randomization	Allocation of mice into groups was random, with exception that age cohorts were evenly distributed across experimental groups and each group contained both male and female (in separated cages) to ensure similar sex proportion among all groups.
Blinding	Investigators were not blinded during data collection or analysis because experiment planning, conducting, and analysis were typically performed by same personnel.

Reporting for specific materials, systems and methods

We require information from authors about some types of materials, experimental systems and methods used in many studies. Here, indicate whether each material, system or method listed is relevant to your study. If you are not sure if a list item applies to your research, read the appropriate section before selecting a response.

Materials & experimental systems

n/a	Involved in the study	
☐	☒	Antibodies
☐	☒	Eukaryotic cell lines
☒	☐	Palaeontology and archaeology
☐	☒	Animals and other organisms
☒	☐	Clinical data
☒	☐	Dual use research of concern

Methods

n/a	Involved in the study	
☒	☐	ChIP-seq
☒	☐	Flow cytometry
☒	☐	MRI-based neuroimaging

Antibodies

Antibodies used

rabbit anti-CCHFV NP pAb (IBT Bioservices 04-0011), Alexa-488 goat anti-rabbit secondary antibody (IgG H&L cross adsorbed, Invitrogen A11034; 1:2500), goat anti human IgG antibody conjugated to alkaline phosphatase (Southern Biotech, Birmingham, AL)

Validation

Rabbit anti-CCHFV NP pAb (IBT Bioservices 04-0011); the manufacturers validated using western blot with CCHFV infected Vero cell lysates and HEK293T cell lysates trasnfexted with recombinant CCHFV N protein.
Alexa-488 goat anti-rabbit secondary antibody (IgG H&L cross adsorbed, Invitrogen A11034) (https://www.thermofisher.com/antibody/product/Goat-anti-Rabbit-IgG-H-L-Highly-Cross-Adsorbed-Secondary-Antibody-Polyclonal/A-11034) was validated using four parameters which include absorption, flourescence, microscopy, and purity from their certificate of analysis.
Goat anti human IgG antibody conjugated to alkaline phosphatase (Southern Biotech, Birmingham, AL) validated via ELISA, FLISAS, FC https://resources.southernbiotech.com/techbul/2040.pdf

Eukaryotic cell lines

Policy information about cell lines and Sex and Gender in Research

Cell line source(s)

Expi293 and ExpiCHO cells were obtained directly from Thermo Fischer Scientific.

Authentication

Cell lines came with certificates of analysis from their vendors (Thermo Fisher Scientific), otherwise no further authentication procedures were performed on our end.

Mycoplasma contamination

Cell lines tested negative for mycoplasma contamination via PCR.

Commonly misidentified lines
(See ICLAC register)

N.A.

Animals and other research organisms

Policy information about studies involving animals; ARRIVE guidelines recommended for reporting animal research, and Sex and Gender in Research

Laboratory animals

B6.12952-lfnarltmlAgt/Mmjax mice (male and female, age 37-44 days at time of virus infection) were purchased from Jackson Labs/ MMRRC (#032045-JAX)

Wild animals

No wild animals were used in this study.

Reporting on sex

Experimental groups in mouse studies equally represented both sexes.

Field-collected samples

No field-collected animal samples were used in this study.

Ethics oversight

The mouse study protocol (3102SPEMOUC) was approved by the Institutional Animal Care and Use Committee (IACUC) at the Roybal Campus of the Centers for Disease Control and Prevention (CDC).

Note that full information on the approval of the study protocol must also be provided in the manuscript.
