# Supplementary material for: Structural characterization of protective non-neutralizing antibodies targeting Crimean-Congo hemorrhagic fever virus
Source: Nat Commun. 2022 Nov 26;13:7298. doi: 10.1038/s41467-022-34923-0 (PMC9701186; doi:10.1038/s41467-022-34923-0)
Supplement: Supplementary file 3 — Source Data [file 41467_2022_34923_MOESM3_ESM.pdf]

nature portfolio

Peer Review File

Structural Characterization of Protective Non-Neutralizing Antibodies targeting Crimean-Congo Hemorrhagic Fever Virus

Open Access This file is licensed under a Creative Commons Attribution 4.0 International License, which permits use, sharing, adaptation, distribution and reproduction in any medium or format, as long as you give appropriate credit to the original author(s) and the source, provide a link to the Creative Commons license, and indicate if changes were made. In the cases where the authors are anonymous, such as is the case for the reports of anonymous peer reviewers, author attribution should be to 'Anonymous Referee' followed by a clear attribution to the source work. The images or other third party material in this file are included in the article's Creative Commons license, unless indicated otherwise in a credit line to the material. If material is not included in the article's Creative Commons license and your intended use is not permitted by statutory regulation or exceeds the permitted use, you will need to obtain permission directly from the copyright holder. To view a copy of this license, visit http://creativecommons.org/licenses/by/4.0/.

REVIEWER COMMENTS</B>

Reviewer #1 (Remarks to the Author):

1. 13G8 is a non-neutralizing antibody that however showed protection efficacy in a mouse model. Thus it is unclear how relevant it is to conduct an in-depth study of 13G8 binding epitope with respect to its functionality since it may well be that 13G8 functions through a distinct mechanism unrelated to the epitope specificity, such as Fc-mediated mechanisms. I am not convinced that this entire study provides much useful information for the vaccine design except that this antibody binds to a relatively conserved epitope.

2. The authors did not show any evidence that CC5-17 possesses neutralization activity against any viral subtypes, in vitro or in vivo, except that it binds to an epitope overlapping with that of 13G8. Many antibodies bind to similar epitopes but exhibit vastly different neutralization activities. Comparing 13G8, an antibody with unclear neutralizing mechanisms, with CC5-17, an antibody with no evidence of neutralization, for their structural characteristics is not helpful for either understanding the immunological relevance or facilitating vaccine design, as the authors claimed.

3. Comparison of 13G8 and CC5-17 efficacy in vivo can be complicated by the fact that 13G8 is a mouse antibody while CC5-17 a human antibody, which may render their differential bioactivities due to several reasons, such as in vivo bioavailability, kinetics and even anti-antibody immune response, which could all adversely affect the antibody's in vivo activity. The authors should provide data such as their respective half-life in vivo.

4. I have difficult to understand the reasons of comparing the tertiary structures of Hoti and IbAr10200 with respect to 13G8 binding since 13G8 showed prophylactic potential against both isolates, suggesting that the structure for 13G8 binding is conserved in these two isolates and the analysis provided little useful information. Can the authors clearly explain the rationale for the analysis ?

5. The authors should analyze the tertiary structures of Hoti and Aigai GP38 with mutations at sites 299, 301 and 303 since the Aigai GP38 with these mutations re-established the antibody binding, suggesting that the introduction of a single mutation induced global conformational changes. This structural determination would help determine the critical structural elements that govern the binding of Aigai mutants with 13G8, which will be helpful for vaccine design and for understanding the structural basis for mediating neutralization. Instead, the structural study of Hoti and IbAr10200 is, to me, not quite informative and unnecessary. I suggest that the authors conduct the comparative structural analysis of Hoti and Aigai mutant binding 13G8.

6. As for CC5-17 analysis, the key caveat is that none of the binding analysis of CC5-17 binding mutant GP38s was simultaneously corroborated by neutralization data. Therefore, it is unclear how relevant and meaningful these data are related to the neutralization property of CC5-17, and how these information would further support CCHFV vaccine design. The authors should clearly explain in the discussion.

7. As the authors did not provide data on CC5-17 in vitro neutralization in reference to 13G8, it is hard to make reasonable analysis of the structural data presented in the manuscript. I suggest that the authors conduct such study.

Reviewer #2 (Remarks to the Author):

• What are the noteworthy results?

Key results are determining new crystal structures of protective mAbs against the GP38 protein of a significant human pathogen, CCHFV, and isolation/characterization of yet more novel anti-GP38 Abs from survivors. Access to new antibodies, molecular-level details of antibody specificity, additional structural/binding information, and in vivo analyses of antibody efficacy all advance basic understanding of immune responses to CCHFVs and provide useful input into design/selection of therapeutic antibodies and potential vaccine immunogens. Therefore, overall impact is real and the manuscript merits publication. The overall presentation is clear and accessible, fairly concise and focused.

The disappointing aspect of this work is that, after considerable effort and thoughtful analysis, few answers are actually forthcoming, including the biological function of GP38 and the mechanism of protection. However, the results as presented represent an important step towards answering these questions.

• Will the work be of significance to the field and related fields?

The results have significant direct impact in-field and some real impact in general on virology and vaccinology more generally.

• Does the work support the conclusions and claims, or is additional evidence needed?

The presented experimental results adequately support the stated conclusions, though the effort to draw as much perceived impact from the consensus of the observations is palpable.

• Are there any flaws in the data analysis, interpretation and conclusions? Do these prohibit publication or require revision?

In general, experimental design/execution/interpretation are sound and rigorous. The crystal structures are fairly low resolution, of average quality for this range, but the authors get a lot of credit for appropriately interpreting these fairly low-resolution structural results with a minimum of over interpretation.

I think it would be useful to add an estimate of coordinate error to Supp. Table 1 for the crystal structures, particularly when reporting low-resolution structures.

However, one glaring deficiency is the total lack of error estimates for all the reported quantitative measurements – this needs to be fixed before publication.

• Is the methodology sound? Does the work meet the expected standards in your field?

Yes and yes – except for the lack of error estimates.

• Is there enough detail provided in the methods for the work to be reproduced?

Yes.

Reviewer #3 (Remarks to the Author):

In this manuscript Durie, Tehrani and colleagues characterise two "site I" antibodies targeting gp38: 13g8 and cc5-17. They obtain the structures of the Ag/Ac complexes, measure their binding affinities to gp38 of different strains and identify escape mutants. The study is original because, as the authors point out, it is the first time that structures of complexes formed by gp38 and protective antibodies are reported. Overall the article is well written and easy to read and the experimental data support almost all of its conclusions. The only part that I disagree with is in lines 240-243 and 271-173 where the authors mention that "the current though that improving binding affinity can correspondingly increase efficacy is not supported by these data, raising new questions about the characteristics needed for non-neutralising mAbs while indicating that binding affinity is not a sole determinant of protection". Although I agree that there are other factors influencing antibody activity, such as avidity or effector functions, which are often linked to the molecular mechanism of protection, which we do not know in

this case. The data presented here do not show that. They are comparing two different antibodies, with two different modes of binding and perhaps even two different mechanisms of protection. In a colloquial way, they are comparing apples and oranges. If they want to show that, for these antibodies, affinity and activity are not correlated, the experiment they should do is to mutate one of these antibodies (to have more or less affinity) and show that this mutated antibodes has the same activity as the WT. Therefore, I think you should rephrase this statement. Otherwise, I have only a few minor comments which I will list below.

MINOR COMMENTS

Line 48. Please include a reference for the classification of CCHFV strains.I would be interested to know if this classification, which I imagine has been done using the RNA polymerase sequence, is congruent with the gp38 sequences, i.e. if using gp38 or the M segment would also give the same clades.

Lines 54 and 69. It has been suggested that gp38 may remain associated with Gn on the particle and on the viral surface (PMID 34960636). In the same reference there is a prediction of the structure of Gn from CCHFV that shows that it has the same folding as the other Gn proteins from bunyaviruses and that gp38 and gn do not arise from a gene duplication event. All these is also relevant for the discussion.

Line 112. Site I epitope is mentioned here but the competition experiment is described later so it is difficult to understand what the site I is.

Lines 114-117. According to the nomenclature used in the literature, this antibody binds to the alpha-helical bundle of gp38.

We thank the reviewers for their contributions towards further strengthening the manuscript. Below is our point-by-point response to address their concerns and suggestions. Our responses are in blue.

REVIEWER COMMENTS
Reviewer #1 (Remarks to the Author):

1. 13G8 is a non-neutralizing antibody that however showed protection efficacy in a mouse model. Thus, it is unclear how relevant it is to conduct an in-depth study of 13G8 binding epitope with respect to its functionality since it may well be that 13G8 functions through a distinct mechanism unrelated to the epitope specificity, such as Fc-mediated mechanisms. I am not convinced that this entire study provides much useful information for the vaccine design except that this antibody binds to a relatively conserved epitope.

CCHFV is among the growing number of viruses for which eliciting neutralizing antibodies is not critical for vaccine efficacy. Our data provides key advances in the field by identifying for the first time the location of a GP38 epitope targeted by protective non-neutralizing mAbs. In addition, we show that affinity for this epitope beyond the threshold of c13G8 does not contribute to greater mAb efficacy as previously supposed. Instead, the orientation of the mAb engaging this epitope on GP38 appears to be the driving factor.

Regarding Fc-mediated mechanism considerations, we would like to highlight that for these studies both CC5-17 and c13G8 have the identical human IgG1 Fc allowing us to propose that the angle at which mAbs are binding to the epitope are important for protection. We thank the reviewer for pointing out that our methodology can be clarified. In addition, the contribution of the Fc in protection by 13G8 and c13G8 appears to only play a partial role in protection, as an Fc effector function knockout variant of c13G8 (c13G8$^{LALAAPG-N}$) still offers some level of protection and Fc knockout mice are still protected with 13G8 treatment (https://doi.org/10.1128/JVI.02005-19, DOI: 10.1126/sciadv.aaw9535). As reviewers 2 and 3 point out, since these are the first GP38-mAb structures described, these data give a clear step forward towards therapeutic development and understanding the immune response to CCHFV.

To address the reviewer's comments and improve clarity of the manuscript, we have made changes to the following sections:

1. *In Materials and Methods*
    a. *Old "The antibodies c13G8 and CC5-17 were expressed by transient transfection of expiCHO-S cells"*
    b. *Revised "Plasmids of c13G8 and CC5-17 were standardized to a human IgG1 Fc backbone and produced by transfection of expiCHO-S cells."*
2. *Subheading: Affinity of non-neutralizing antibodies to site-I is not the sole driver of protective efficacy*
    a. *Old "To investigate if increased binding affinity of CC5-17 confers higher protective efficacy than c13G8, we evaluated clinical course and outcome*

following treatment in the IFNAR$^{-/-}$ mouse model of lethal disease. Groups of mice (n = 4–6 per group) were injected subcutaneously with a target dose of 100 $TCID_{50}$ of CCHFV Turkey-2004 and subsequently treated intraperitoneally with mAb or isotype control.

b. *Revised "To investigate if increased binding affinity of CC5-17 confers higher protective efficacy than c13G8, we evaluated clinical course and outcome following treatment in the IFNAR$^{-/-}$ mouse model of lethal disease. Groups of mice (n = 4–6 per group) were infected subcutaneously with a target dose of 100 $TCID_{50}$ of CCHFV Turkey-2004 and subsequently treated intraperitoneally with standardized IgG1 site I mAbs or isotype IgG1 control."*

3. *Figures 1,2,3 were updated to clarify the use of c13G8.*

2. The authors did not show any evidence that CC5-17 possesses neutralization activity against any viral subtypes, in vitro or in vivo, except that it binds to an epitope overlapping with that of 13G8. Many antibodies bind to similar epitopes but exhibit vastly different neutralization activities. Comparing 13G8, an antibody with unclear neutralizing mechanisms, with CC5-17, an antibody with no evidence of neutralization, for their structural characteristics is not helpful for either understanding the immunological relevance or facilitating vaccine design, as the authors claimed.

The literature surrounding GP38 supports that antibodies that bind to GP38, including 13G8, are non-neutralizing (Golden et al. 2019). We thank the reviewer for their suggestion to provide evidence of the non-neutralizing activity for the newly identified human mAbs targeting GP38 to ensure the mechanism in these human mAbs match that of their mouse counterparts. To this end, we illustrated that c13G8, CC5-17 and all the human GP38 mAbs isolated were not neutralizing. We have edited the manuscript to include these data in Supplementary Figure 4.

*Within subheading of "*Characterization of human derived anti-GP38 mAbs" *we added the statement "Additionally, human GP38 mAbs from CC5 failed to neutralize CCHFV infection, suggesting that antibody response toward GP38 is mainly or exclusively non-neutralizing (Supplementary, Figure 4)." Additionally, from our neutralization data we can conclude that non-neutralizing mAbs that bind the N-terminal helical region are non-neutralizing and can protect mice from CCHFV challenge providing groundwork for the rational design of vaccine antigens.*

3. Comparison of 13G8 and CC5-17 efficacy in vivo can be complicated by the fact that 13G8 is a mouse antibody while CC5-17 a human antibody, which may render their differential bioactivities due to several reasons, such as in vivo bioavailability, kinetics and even anti-antibody immune response, which could all adversely affect the antibody's in vivo activity. The authors should provide data such as their respective half-life in vivo.

We agree that if the antibodies being compared had different species-specific Fc regions (one human and one mouse) as interpreted by the reviewer, that additional characterization and comparisons would be warranted.

However, while 13G8 was initially identified in mice and CC5-17 in humans, for the purpose of these experiments both antibodies were on the human IgG1 Fc and thus differences in origin of antibody sequence should not affect kinetics in vivo. Furthermore, there is precedence in the literature to support the use of the human chimeric in murine protection studies, and both their and our efficacy data demonstrate comparable results to those obtained with mouse 13G8 (PMID: 31996434). In addition, our animal studies were in the IFNAR -/- mouse model of acute lethal disease which succumbs to disease in ~6-8 days. Two doses of the mAb were given three days apart and the antibody half-life for IgG1 in mice has been observed to be around 6-8 days (PMID: 9036967, 16139891, 3350037), and thus would not have substantial influence on the outcome in this model.

We thank the reviewer for their comment and for highlighting the need to clarify the experimental approach used to rule out potentially confounding effects of Fc region differences. The methods and figure legends were edited accordingly for clarity. Please see response to Reviewer 1, point #1.

4. I have difficult to understand the reasons of comparing the tertiary structures of Hoti and IbAr10200 with respect to 13G8 binding since 13G8 showed prophylactic potential against both isolates, suggesting that the structure for 13G8 binding is conserved in these two isolates and the analysis provided little useful information. Can the authors clearly explain the rationale for the analysis?

Prior to this study, the GP38 structure was only characterized from the IbAr10200 strain, which is a different clade than Hoti, and a strain that is viewed by the field as a highly passaged isolate (IbAr10200). This left open the question of whether GP38s from different clades or more relevant clinical strains had tertiary structural changes that may impact the ability of mAbs to access certain epitopes. As seen, overall, there is a high similarity between the two structures. We thank the reviewer for pointing out that further clarification can be added. The following change was made within subheading "Structure of a GP38 from CCHFV strain Hoti":

Original: "With c13G8 showing nanomolar or better affinity towards various CCHFV strains, but not those of the Aigai virus, the molecular factors behind this phenomenon were sought out. To provide the most clinical relevance to the study, the CCHFV circulating strain Hoti was used. This strain is one of the two that can be used to elicit human disease-like symptoms in a non-human primate model [16-21].

Revised: "With c13G8 showing nanomolar or better affinity towards various CCHFV strains, but not those of the Aigai virus, the molecular factors behind this phenomenon were sought out. As only one structure of GP38 has been solved from clade III CCHFV IbAr10200, a strain with little clinical relevance, we solved an additional structure of GP38 from Clade V CCHFV Hoti. This strain is one of the two that can be used to elicit human disease-like symptoms in a non-human primate model[16-21].

5. The authors should analyze the tertiary structures of Hoti and Aigai GP38 with mutations at

sites 299, 301 and 303 since the Aigai GP38 with these mutations re-established the antibody binding, suggesting that the introduction of a single mutation induced global conformational changes. This structural determination would help determine the critical structural elements that govern the binding of Aigai mutants with 13G8, which will be helpful for vaccine design and for understanding the structural basis for mediating neutralization. Instead, the structural study of Hoti and IbAr10200 is, to me, not quite informative and unnecessary. I suggest that the authors conduct the comparative structural analysis of Hoti and Aigai mutant binding 13G8.

This point was partially addressed above (Reviewer 1, point #4), with the central desire to obtain the most clinically relevant information for CCHFV countermeasure development by obtaining information on the GP38 from the Hoti strain. In terms of the comment regarding neutralization, we would like to clarify, as we have in earlier points, that these mAbs are non-neutralizing mAbs.

Naturally one of the benefits of being able to now compare GP38 structures originating from IbAr10200 and Hoti (as seen in Figure S1,2,3c), is the further confirmation that these mutational sites in question are all observed as bulk water facing located within a conserved alpha helix when a mAb is not bound. This alpha helix is predicted with high confidence to be there with or without these mutations across GP38s found in CCHFV and Aigai virus (Jpred). This is in line with the literature which highlights that single point mutation as the ones employed here are well tolerated in alpha helices (PMID 35412967). As mentioned in the manuscript, this is level of conservation for site I is not a surprise given the location of the epitope includes the host furin cleavage site. A site where tertiary structure changes would be selected against because they would impact the viral fitness.

As described in the manuscript, the mutational studies at the locations in question were to classically confirm whether those specific positions highlighted by the X-ray structural information play a part in the ability for the protective c13G8 and CC5-17 to engage GP38 and if so, how much? As good as any structure-function relation seen in structural biology, the mutations mirrored the expected results arising by observations with the mAb-GP38 complex and that of the Aigai homology model as seen in Figure 4c providing this insight. In short, the mutations at positions that would appear to cause major difficulties with the binding of one mAb (CC5-17) were as predicted not observed in the other c13G8 and vice versa. Hence, additional of X-ray structures with GP38s containing non-naturally occurring mutations would be of limit benefit from a clinical or biochemical prospective as the experimentally determine structures and models arising from them already provide the information.

6. As for CC5-17 analysis, the key caveat is that none of the binding analysis of CC5-17 binding mutant GP38s was simultaneously corroborated by neutralization data. Therefore, it is unclear how relevant and meaningful these data are related to the neutralization property of CC5-17, and how this information would further support CCHFV vaccine design. The authors should clearly explain in the discussion.

The neutralization comment is addressed in more detail above (Reviewer 1, point #2).

We added the following to better explain how this work could support CCHFV vaccine design:

"The characterization of this site provides a rationale for the development of vaccines and mAbs targeting GP38 site I, in addition this site can be utilized for future vaccination methods to fully cover the whole spectrum of CCHFV genetic diversity (clades I-V) and potentially other nairoviruses."

7. As the authors did not provide data on CC5-17 in vitro neutralization in reference to 13G8, it is hard to make reasonable analysis of the structural data presented in the manuscript. I suggest that the authors conduct such study.

We addressed the reviewer's concern by including new data showing that GP38 mAbs studied are non-neutralizing. Further details regarding this comment are provided above (Reviewer 1, point #2).

Reviewer #2 (Remarks to the Author):

1. What are the noteworthy results?
Key results are determining new crystal structures of protective mAbs against the GP38 protein of a significant human pathogen, CCHFV, and isolation/characterization of yet more novel anti-GP38 Abs from survivors. Access to new antibodies, molecular-level details of antibody specificity, additional structural/binding information, and in vivo analyses of antibody efficacy all advance basic understanding of immune responses to CCHFVs and provide useful input into design/selection of therapeutic antibodies and potential vaccine immunogens. Therefore, overall impact is real and the manuscript merits publication. The overall presentation is clear and accessible, fairly concise and focused.

The disappointing aspect of this work is that, after considerable effort and thoughtful analysis, few answers are actually forthcoming, including the biological function of GP38 and the mechanism of protection. However, the results as presented represent an important step towards answering these questions.

We thank the reviewer for acknowledging the significance of our results and shared enthusiasm towards elucidating GP38 function using antibodies and structure presented herein.

2. Will the work be of significance to the field and related fields?
The results have significant direct impact in-field and some real impact in general on virology and vaccinology more generally.

We thank the reviewer for the positive feedback on the broad scientific impact of this manuscript.

3. Does the work support the conclusions and claims, or is additional evidence needed? Are there any flaws in the data analysis, interpretation and conclusions? Do these prohibit publication or require revision?

In general, experimental design/execution/interpretation are sound and rigorous. The crystal structures are fairly low resolution, of average quality for this range, but the authors get a lot of credit for appropriately interpreting these fairly low-resolution structural results with a minimum of over interpretation.

I think it would be useful to add an estimate of coordinate error to Supp. Table 1 for the crystal structures, particularly when reporting low-resolution structures.

We appreciate the thoughtful comments from Reviewer 2 and added an estimate of coordinate error to Supp. Table 1 within the supplementary methods. This data point is in line with other structures of these resolutions.

However, one glaring deficiency is the total lack of error estimates for all the reported quantitative measurements – this needs to be fixed before publication.

We thank the reviewer for this comment and have updated the report to include these data where appropriate. In regards to this issue when working with the BLI, the binding kinetics are done by a 1:1 binding model over several concentrations and curves are fit and at each concentration. The KD is then derived globally from the concentration range and the error given is based on how it fits the binding model. We now include this information in the manuscript. Added within methods subheading "BLI Kinetics Assay mAb to GP38"

"At each concentration one curve was performed for n=1."

Apart from the BLI kinetics, the ELISA graphs in Figure 2A/B were fixed to include error bars representing the standard deviation of replicates (n=2) and the methodology now includes this information.

4. Is the methodology sound? Does the work meet the expected standards in your field?
Yes and yes – except for the lack of error estimates.

Error bars were added to the figures as needed.

5. Is there enough detail provided in the methods for the work to be reproduced?
Yes.

Reviewer #3 (Remarks to the Author):

In this manuscript Durie, Tehrani and colleagues characterize two "site I" antibodies targeting gp38: 13g8 and cc5-17. They obtain the structures of the Ag/Ac complexes, measure their binding affinities to gp38 of different strains and identify escape mutants. The study is original because, as the authors point out, it is the first time that structures of complexes formed by gp38 and protective antibodies are reported. Overall, the article is well written and easy to read and the experimental data support almost all of its conclusions. The only part that I disagree with is in lines 240-243 and 271-173 where the authors mention that "the current though that improving

binding affinity can correspondingly increase efficacy is not supported by these data, raising new questions about the characteristics needed for non-neutralizing mAbs while indicating that binding affinity is not a sole determinant of protection". Although I agree that there are other factors influencing antibody activity, such as avidity or effector functions, which are often linked to the molecular mechanism of protection, which we do not know in this case. The data presented here do not show that. They compare two different antibodies, with two different modes of binding and perhaps even two different mechanisms of protection. In a colloquial way, they compare apples and oranges. If they want to show that, for these antibodies, affinity and activity are not correlated, the experiment they should do is to mutate one of these antibodies (to have more or less affinity) and show that these mutated antibodies have the same activity as the WT. Therefore, I think you should rephrase this statement. Otherwise, I have only a few minor comments which I will list below.

We thank the reviewer for the constructive comments and for noting the merit of this manuscript. The reviewer brings up a valuable point on the statement we made about binding affinity and the interaction of site-I mAbs. Our intention was to build upon previous research (Golden et al. 2019) suggesting that increased binding affinity of 13G8 over another site-I mAb, 10E11, might explain why only 13G8 is effective at protecting mice from CCHFV challenge. Therefore, we tested this hypothesis by comparing CC5-17, an antibody showing increased binding affinity compared to 13G8 or c13G8. Treatment of mice with lower affinity c13G8 was proven more efficacious than CC5-17 showing improved binding kinetics. Therefore, we demonstrate for the first time that improved binding to site-I epitope is not directly correlating with protection. Instead, the angle at which c13G8 engages the epitope might be a more significant contributing factor to GP38 mAb efficacy.

The following changes were made to address the reviewer's concern and the text was edited as suggested to clarify our previous statements in regards to mAb affinity vs. protection:

1. *Section 240-243*
    a. *Old "The path to establish a more efficacious mAb through competition assays and binding affinity is complex, as seen here with CC5-17 The current thought that improving binding affinity can correspondingly increase efficacy is not supported by these data, raising new questions about the characteristics needed for non-neutralizing mAbs while indicating that binding affinity is not a sole determinant of protection."*
    b. *Revised "The path to establish a more efficacious mAb through competition assays and binding affinity is complex, as seen here with CC5-17, in which antibodies targeting the same site can have significant different efficacy, which raises new questions about the characteristics needed for anti-GP38 to provide protection.*
2. *Section 271-273*

a. *Old "While this highlights that affinity is not the sole driver of efficacy for non-neutralizing anti-GP38 mAbs, it also suggests that other factors are likely at play."*
b. *Revised "Previous research discussed the possibility that binding affinity could be a determinant of efficacy towards site I. Through our studies, site I mAbs can have drastically different efficacy and further exploration needs to be investigated towards the drivers of protection apart from binding affinity.*

MINOR COMMENTS

1. Line 48. Please include a reference for the classification of CCHFV strains. I would be interested to know if this classification, which I imagine has been done using the RNA polymerase sequence, is congruent with the gp38 sequences, i.e., if using gp38 or the M segment would also give the same clades.

A reference was added in the noted section regarding the classification of CCHFV strains.

2. Lines 54 and 69. It has been suggested that gp38 may remain associated with Gn on the particle and on the viral surface (PMID 34960636). In the same reference there is a prediction of the structure of Gn from CCHFV that shows that it has the same folding as the other Gn proteins from bunyaviruses and that gp38 and gn do not arise from a gene duplication event. All this is also relevant for the discussion.

Thank you for the suggestions. PMID 34960636 was added as a reference, additionally speculation of the GP38/Gn relationship was added in the introduction within those lines to further describe the potential relationship between GP38 and GN and clarification.

Original "While the role of GP38 is not very well understood, a recent X-ray structure of a laboratory isolate, strain IbAr10200, has spurred speculation of GP38 originating from a G_N gene duplication event"

Revised "While the role of GP38 is not fully understood, there is speculation that GP38 is still attached to the G_N and does not originate from gene duplication event"

In the discussion the following paragraph was expanded and now reads as follows:

"Of interest structurally and biochemically is that both c13G8 and CC5-17 partially bind to the N-terminus near the furin cleavage site. Through processing of the CCHFV GPC, GP160 and GP85 are further cleaved by furin to yield the free GP38 and MLD. The proximity of the site I antibodies epitope with furin cleavage suggests that inhibition of processing might contribute to mAb efficacy as this cleavage contributes to CCHFV infectivity [13]. In addition, furin could cleave immature forms of Pre-Gn upon viral entry if associated with the virion as observed for the uncleaved surface glycoproteins of coronaviruses and flaviviruses [41,42]. If efficacy of GP38 mAbs were limited to those binding the N-terminus capable of blocking furin processing, this would focus future mAbs development efforts toward this specific activity."

3. Line 112. Site I epitope is mentioned here but the competition experiment is described later so it is difficult to understand what the site I is.

Site I was taken out of the sentence to assist in reader comprehension.

Original "The electron density around the binding interface is well defined, and the c13G8 site I epitope can be clearly identified (Supplementary Figure 4a)"

Revised "The electron density around the binding interface is well defined, and the c13G8 epitope can be clearly identified (Supplementary Figure 4a)"

4. Lines 114-117. According to the nomenclature used in the literature, this antibody binds to the alpha-helical bundle of gp38.

This nomenclature was used to further describe the binding interaction

Original "Two main components of this interface are also identified"

Revised "Overall c13G8 engages with the alpha-helical bundle of GP38 that are characterized through two main components"

REVIEWERS' COMMENTS

Reviewer #3 (Remarks to the Author):

The authors have responded satisfactorily to all my comments. I congratulate them for their work.
